# A five metastasis-related long noncoding RNA risk signature for osteosarcoma survival prediction

**DOI:** 10.1186/s12920-021-00972-5

**Published:** 2021-05-08

**Authors:** SiYuan Yu, FengLing Shao, HuiJun Liu, QingQing Liu

**Affiliations:** 1 Department of Anesthesiology, Children’s Hospital of Chongqing Medical University, National Clinical Research Center for Child Health and Disorders, Ministry of Education Key Laboratory of Child Development and Disorders, Chongqing, China; 2 Department of Pediatric Surgical Oncology, Children’s Hospital of Chongqing Medical University, National Clinical Research Center for Child Health and Disorders, Ministry of Education Key Laboratory of Child Development and Disorders, Chongqing, China; 3 Chongqing Key Laboratory of Pediatrics, Chongqing, China; 4 Reproductive Medical Center of Gansu Provincial Maternal and Child-Care Hospital, Lanzhou, China

**Keywords:** Long noncoding RNA, Bioinformatics analysis, Prognostic biomarker, Osteosarcoma, TARGET database

## Abstract

**Background:**

Osteosarcoma is a highly malignant and common bone tumour with an aggressive disease course and a poor prognosis. Previous studies have demonstrated the relationship between long noncoding RNAs (lncRNAs) and tumorigenesis, metastasis, and progression.

**Methods:**

We utilized a large cohort from the Therapeutically Applicable Research to Generate Effective Treatments (TARGET) database osteosarcoma project to identify potential lncRNAs related to the overall survival of patients with osteosarcoma by using univariate and multivariate Cox proportional hazards regression analyses. Kaplan–Meier curves were generated to evaluate the overall survival difference between patients in the high-risk group and the low-risk group. A time-dependent receiver operating characteristic curve (ROC) was employed, and the area under the curve (AUC) of ROC was measured to assess the sensitivity and specificity of the multi-lncRNA signature.

**Results:**

Five lncRNAs (RP11-128N14.5, RP11-231|13.2, RP5-894D12.4, LAMA5-AS1, RP11-346L1.2) were identified, and a five-lncRNA signature was constructed. The AUC for predicting 5-year survival was 0.745, which suggested good performance of the five-lncRNA signature. In addition, functional enrichment analysis of the five-lncRNA-correlated protein-coding genes (PCGs) was performed to show the biological function of the five lncRNAs. Additionally, PPI network suggested RTP1 is a potential biomarker that regulates the prognosis of osteosarcoma.

**Conclusions:**

We developed a five-lncRNA signature as a potential prognostic indicator for osteosarcoma.

**Supplementary Information:**

The online version contains supplementary material available at 10.1186/s12920-021-00972-5.

## Background

Osteosarcoma is a highly malignant and common bone tumour with an aggressive and poor prognosis and is prevalent in adolescents and young adults [1, 2]. Approximately 80% of osteosarcomas occur in the extremities, most commonly in the metaphysis of the long bone around the knee joint, and the other 20% occur in the axial bone and pelvis [3]. In addition, metastasis, especially lung metastasis, is an important factor in determining the prognosis and survival of osteosarcoma. It has been reported that high-dose methotrexate, vincristine, and folinic acid could improve the survival rate of patients. However, the survival rate of patients with metastatic osteosarcoma remains unchanged [4]. Therefore, we aimed to search for prognostic targets of osteosarcoma.

The function of long noncoding RNAs (lncRNAs) in many areas has gradually gained an appreciation and has been extensively studied. lncRNAs are defined as transcripts of more than 200 nucleotides that are not translated into proteins [5] and can participate in transcription, post-transcriptional regulation, and protein regulation. Findings from numerous epidemiological studies have linked lncRNAs to tumours, which suggests that lncRNAs play an important role as biomarkers [6, 7] and therapeutic targets [8, 9]. In addition, the role of lncRNAs in osteosarcoma is currently of significant interest to many researchers. Previous studies have demonstrated the relationship between lncRNAs and tumorigenesis [9, 10], metastasis [11], progression [12], and chemotherapeutic drug resistance [13] in osteosarcoma. Unfortunately, there is limited intervention research on the impact of potential prognostic lncRNA signatures for osteosarcoma.

The Therapeutically Applicable Research to Generate Effective Treatments (TARGET) database provides RNA-sequencing (mRNA-seq) data on several types of cancers, including acute lymphoblastic leukaemia, myeloid leukaemia, kidney tumours, neuroblastoma, and osteosarcoma. In this study, a large cohort from the TARGET osteosarcoma project was used to identify potential lncRNAs related to the overall survival of patients with osteosarcoma. In addition, we identified a five-lncRNA signature that could predict the prognosis of patients with osteosarcoma. Our study suggests the potential functions of lncRNAs in osteosarcoma.

## Methods

### Data collection and preprocessing

Data of mRNA-seq and relevant clinical sample data of osteosarcoma patients were collected through the TARGET data matrix (https://ocg.cancer.gov/programs/target). We downloaded data for 306 clinical samples in total, but only 102 clinical samples had mRNA-seq data, and after deleting duplicate data, there were only 97 samples. Altogether, 24 metastatic samples and 73 non-metastatic samples were used to determine differentially expressed lncRNAs by using the edger package in R software (4.0.2). Differently expressed lncRNAs were selected based on threshold log foldchange (FC) > 1, adjusted *p* < 0.05.

### Identification of a prognostic lncRNA signature

We excluded patients without vital status and lncRNA sequence data. A total of 95 patients (providing 23 metastatic samples and 72 non-metastatic samples) were concluded in the research. Then, univariate Cox proportional hazards regression analysis was used to select significant lncRNAs related to the survival status (Additional file 1: Table S1). Finally, we identified 5 lncRNAs (RP11-128N14.5, RP11-231|13.2, RP5-894D12.4, LAMA5-AS1, RP11-346L1.2) according to *P* < 0.05 and HR > 1 and using R software (4.0.2).

### Independence of the multi-lncRNA signature for predicting the prognosis of patients with osteosarcoma

To construct the prognostic multi-lncRNA signature, we conducted multivariate Cox proportional hazards regression analysis. A risk score was calculated by integrating the lncRNA expression profiles and relative multivariate Cox regression coefficients (Additional file 2: Table S2). According to the risk score, patients with osteosarcoma consisted of a group with low-risk and a group with high-risk. Then, Kaplan–Meier curves were generated to estimate the whole surviving discrepancy among invalids in the team with high-risk and team with low-risk. In addition, a receiver that was time-oriented and operated characteristic curve (ROC) was adopted to identify specificity and sensitivity of multi-lncRNA signature with the help of measuring region under the curve of ROC [14, 15].

### Functional enrichment analysis

lncRNA-correlated protein-coding genes (PCGs) was the base of the analysis of functional enrichment. A Pearson analysis of connection for every prognostic lncRNAs of PCGs according to sequence data of TARGET data matrix (Additional file 3: Table S3) was launched. PCGs about Pearson coefficients > 0.5 were reckoned to be lncRNA-related PCGs. Next, the pathway analysis of gene ontology (GO) and Kyoto Encyclopedia of Genes and Genomes (KEGG) was operated through the database aimed for annotation, visualization, and integrated discovery (http://metascape.org/) Internet tool [16]. Biological process (BP) terms and KEGG pathways of *P* < 0.05 were thought to be important.

### Protein–protein interaction network construction

Protein–Protein interaction (PPI) analysis of lncRNA-correlated PCGs was constructed on the STRING protein database 11.0 (http://string-db.org/). We set the interaction score > 0.4 as the cut-off criterion[17, 18].

## Results

### Resource identification initiative statement

We downloaded 306 clinical samples, but only 102 clinical samples had mRNA-seq data, and after deleting duplicate data, only 97 samples remained. After excluding patients without vital status and lncRNA sequence data, 95 patients (providing 23 metastatic samples and 72 non-metastatic samples) were included in this study. Specific information is shown in Table 1.

**Table 1:** Clinicopathologic characteristics of patients with osteosarcoma

Characteristics	Sample size	Ratio (%)
Sex		
Female	39	41.1
Male	56	58.9
Age, y		
≤ 18	73	76.8
> 18	22	23.2
Disease state at diagnosis		
Metastatic	23	24.2
Non-metastatic	72	75.8
Vital status		
Alive	57	60
Dead	38	40

### Identification of differentially expressed lncRNAs

**Fig. 1:**
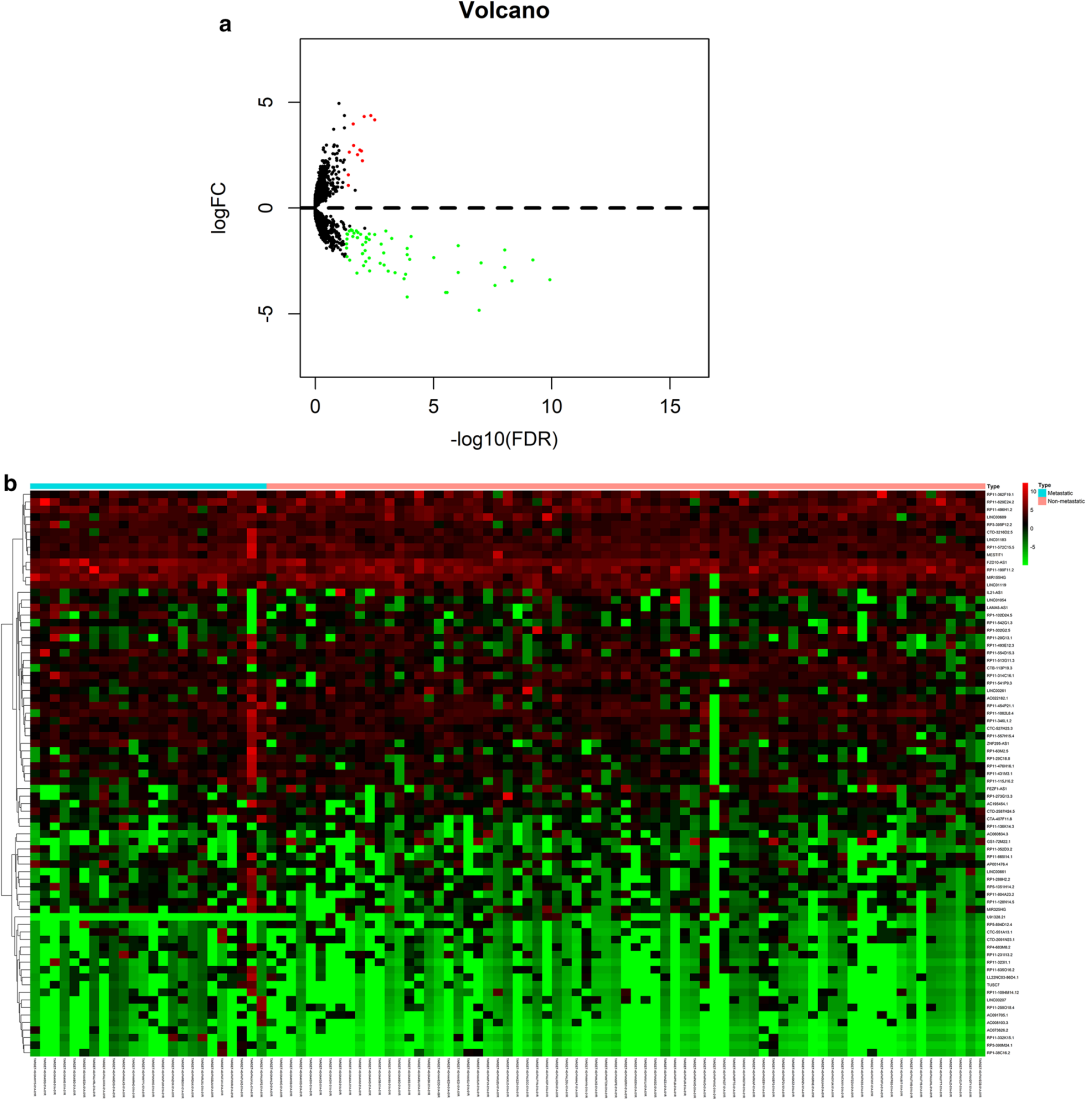
**a** Volcano plot of differentially expressed long noncoding RNAs (lncRNAs) between metastatic osteosarcoma samples and non-metastatic osteosarcoma samples; a total of 75 lncRNAs differentially expressed lncRNAs were identified, including 63 downregulated lncRNAs and 12 upregulated lncRNAs (cut-off criteria: log foldchange(FC) > 1, adjusted *p* < 0.05). Red represents upregulated lncRNAs, and green represents down-regulated lncRNAs. **b** Hierarchical clustering analysis of differentially expressed lncRNAs. Red represents upregulated lncRNAs, and green represents down-regulated lncRNAs. LncRNA, long noncoding RNA

Since differential analysis of expression, about 75 different meaning lncRNAs in total were determined, for example, 63 downregulated lncRNAs and 12 upregulated lncRNAs (cut-off criteria: logFC > 1, adjusted *p* < 0.05). Volcano plot analysis was used to assess variation in lncRNA expression between metastatic samples and non-metastatic samples (Fig. 1a). In addition, hierarchical clustering was used to distinguish these two populations based on lncRNA expression data (Fig. 1b). Then, we identified 5 lncRNAs (RP11-128N14.5, RP11-231|13.2, RP5-894D12.4, LAMA5-AS1, RP11-346L1.2) according to *P* < 0.05 and HR > 1 by using univariate Cox proportional hazards regression analysis.

### Results of the multivariate Cox proportional hazards model

**Fig. 2:**
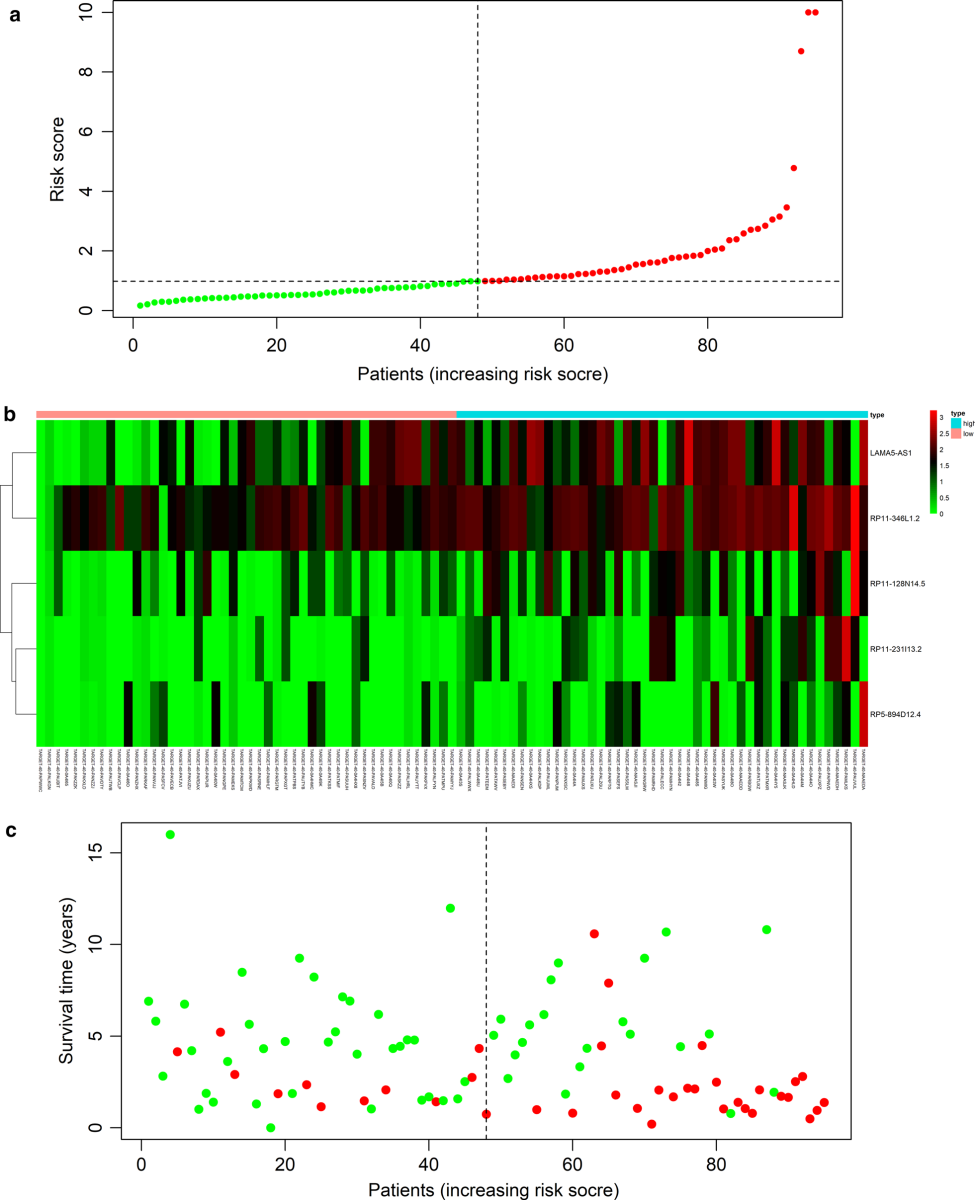
Prognostic value of the five-lncRNA signature in patients with osteosarcoma. Risk score distribution (**a**), expression profiles of the five lncRNAs (**b**), and survival status (**c**)

The multivariate Cox proportional hazards model was established with 5 lncRNAs (RP11-128N14.5, RP11-231|13.2, RP5-894D12.4, LAMA5-AS1, RP11-346L1.2). Multivariate Cox regression analysis was operated on the basis of lncRNA expression and patient survival (Table 2). The expression of RP11-128N14.5, RP11-231|13.2, RP5-894D12.4, LAMA5-AS1, and RP11-346L1.2 was actively connected with survival risk. Osteosarcoma patients were divided into high- and low-risk patient groups according to the score of risk (Fig. 2a). A heatmap of the meaning of the 5 lncRNAs is displayed in Fig. 2b. With the risk score increasing, the expression of RP11-128N14.5, RP11-231|13.2, RP5-894D12.4, LAMA5-AS1, and RP11-346L1.2 increased. The connection between the survival of osteosarcoma patients and the risk score (Fig. 2c) suggested that as the score of risk rose, the risk of death in osteosarcoma patients increased, indicating that high-risk scores osteosarcoma patients have shorter surviving times than low-risk scores patients.

**Table 2:** Multivariate Cox proportional hazards model of the five-lncRNA signature

Gene symbol	Coef	Exp (coef)	SE (coef)	HR	Pr(>|z|)
RP11-128N14.5	0.247734	1.281119	0.130509	1.340221	0.057669
RP11-231|13.2	0.287661	1.333306	0.182852	1.499192	0.115675
RP5-894D12.4	0.294636	1.342637	0.129259	1.470435	0.022642
LAMA5-AS1	0.310018	1.36345	0.11846	1.235036	0.008869
RP11-346L1.2	0.232996	1.262377	0.133438	1.379240	0.080793

### Survival prediction in osteosarcoma patients using the five lncRNAs

**Fig. 3:**
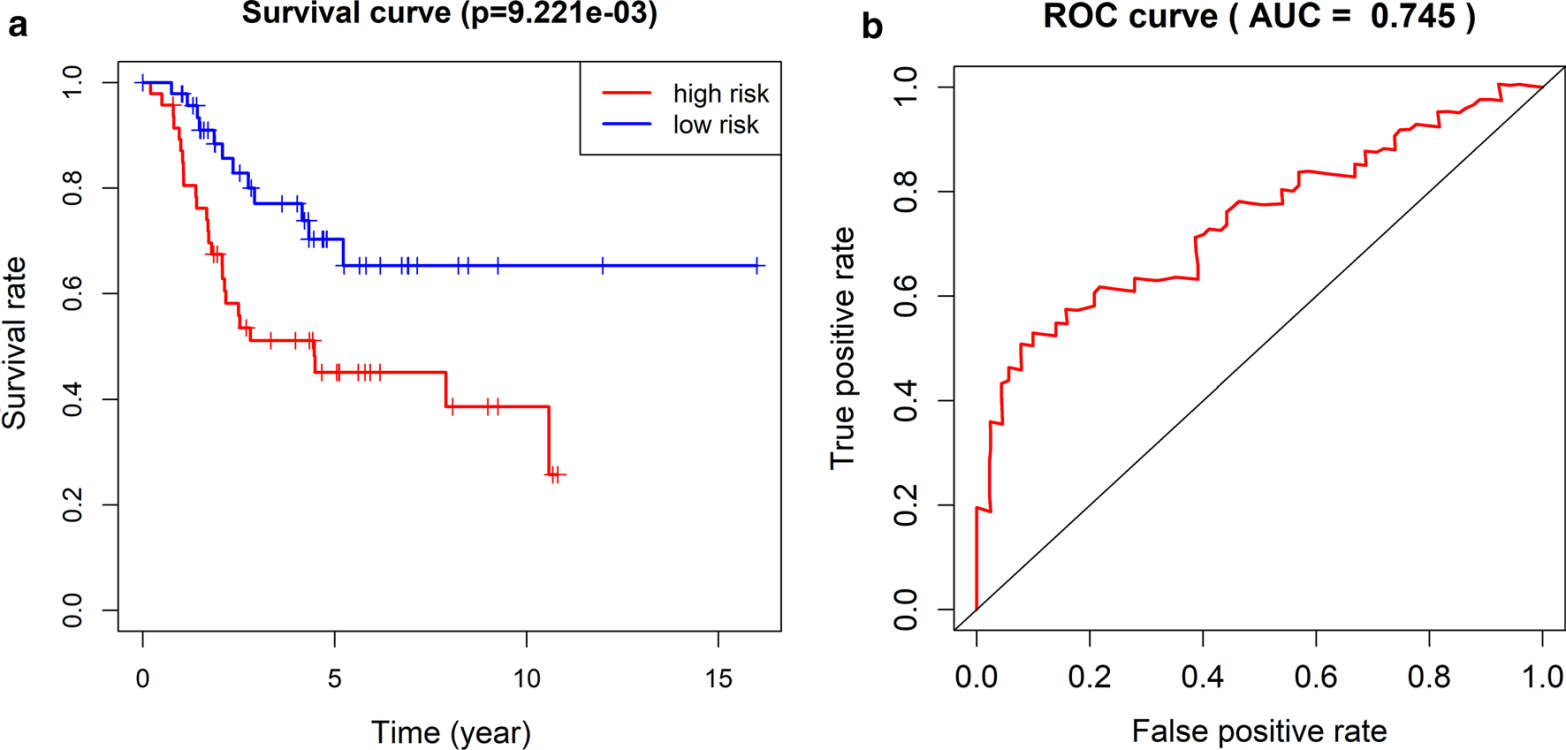
Kaplan–Meier analysis of the overall survival of osteosarcoma patients classified into high-risk and low-risk groups based on the five-lncRNA signature (**a**). Receiver operating characteristic (ROC) curve analysis of the five-lncRNA signature for predicting the 5-year survival of patients with osteosarcoma (**b**). AUC, area under the curve; lncRNA, long noncoding RNA

The multivariate Cox proportional hazards model was built on the basis of the expression of 5 lncRNAs and the survived osteosarcoma patients. The osteosarcoma invalids consisted of high- or low-risk patients according to the score of risk. Next, Kaplan–Meier analysis was utilized to detect the connection between the risk score and patient five-year survival (Fig. 3a). These outcome suggested that the survival rate of high-risk patients was obviously lower than that in with low-risk patients (*p* = 9.221e−03). In addition, we constructed a ROC curve to assess the success of the binomial classification model of high- and low-risk patients (Fig. 3b). The AUC value was 0.745, which suggests that patients can consist of high-risk group and low-risk group, with clear discrepancy in survival, according to risk score. LncRNAs RP11-128N14.5, RP11-231|13.2, RP5-894D12.4, LAMA5-AS1, and RP11-346L1.2 have the potential to estimate the survival of patients with osteosarcoma.

### Functional enrichment analysis

**Fig. 4:**
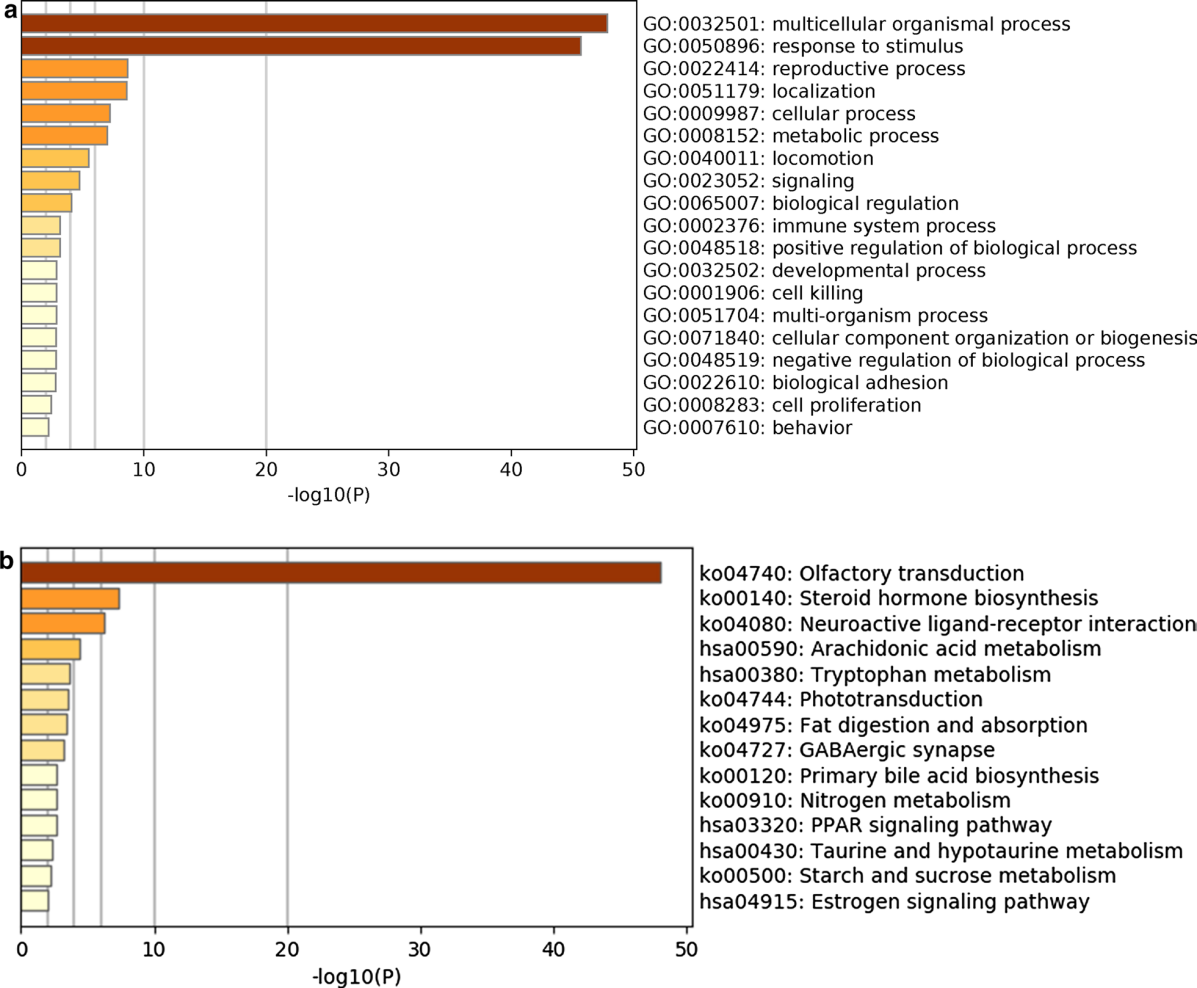
Functional enrichment analysis of the five lncRNAs in the signature based on their correlated protein-coding genes (PCGs). Biological processes (BPs) in the gene ontology (GO) analysis (**a**). Kyoto Encyclopedia of Genes and Genomes (KEGG) pathway analysis (**b**)

950 related PCGs of a Pearson coefficient greater than 0.5 was identified. Final outcome of analysis of functional enrichment showed that the PCGs were mostly rich in BP terms, for example, multicellular organismal process, response to stimulus, reproductive processes, localization, cellular processes, and metabolic processes (Fig. 4a). The KEGG pathway analysis results showed that olfactory transduction, steroid hormone biosynthesis, neuroactive ligand-receptor interaction, and arachidonic acid metabolism were the main associated pathways (Fig. 4b).

### Protein–protein interaction network construction

**Fig. 5:**
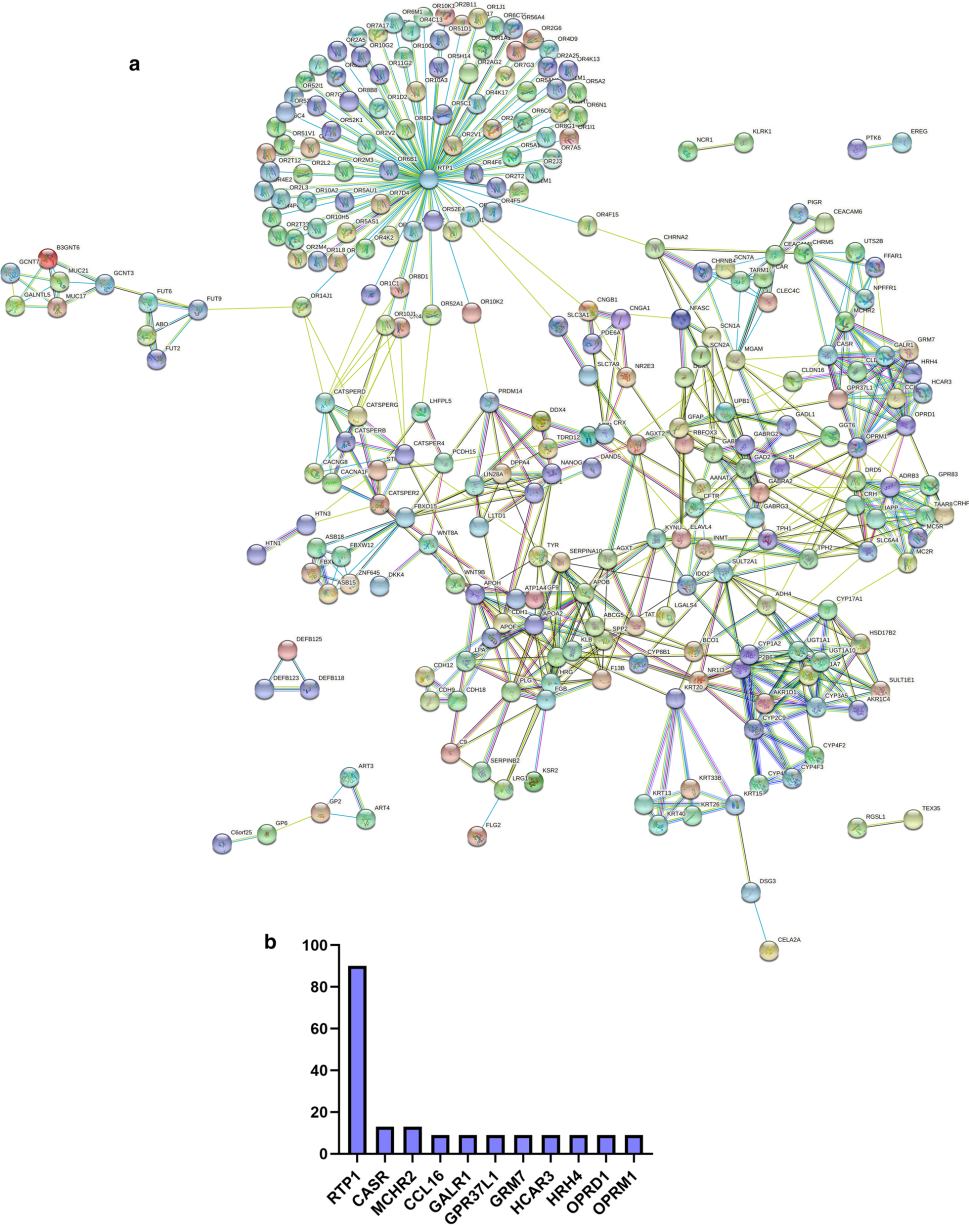
Protein–protein interaction network of lncRNA-correlated PCGs shown by STRING (**a**). Network nodes represent proteins, and edges represent protein–protein associations. The bar plot shows the top 10 ranked proteins (**b**)

The PPI network of lncRNA-correlated PCGs was also employed with the STRING protein database 11.0 (Fig. 5a). The number of nodes was 270, and the number of edges was 663. The average node degree was 4.91 and the average local clustering coefficient was 0.719. We also counted the number of times that each node appeared in the network (Fig. 5b). Finally identified that RTP1 may play a central role in the PPI network.

## Discussion

Osteosarcoma is the most common primary bone tumour, and the five-year survival rate of patients with metastasis is much lower than that of patients without metastasis [19]. Yuan Li et al. [20] demonstrated that the expression of four genes, HIPK2, MAP3K5, CK5, and KRT5, was correlated with survival risk by using a multivariate Cox proportional hazards model. These results show that KRT5, HIPK2, MAP3K5, and CD5 serve as prognostic factors in osteosarcoma patients. In addition, lncRNAs have emerged as potential prognostic biomarkers of tumours. Although many studies have elucidated the relationship between lncRNAs and osteosarcoma [21–23], there is still a lack of knowledge regarding lncRNAs related to survival prediction in patients with osteosarcoma.

In this study, we utilized 95 osteosarcoma samples obtained from the TARGET database that were divided into metastatic and non-metastatic groups, and we analyzed their lncRNA expression profiles. Then, we constructed a lncRNA-based prognostic signature that accounts for the expression of five lncRNAs (RP11-128N14.5, RP11-231|13.2, RP5-894D12.4, LAMA5-AS1, and RP11-346L1.2), which were assumed to be significant according to their multivariate Cox regression coefficients. Unfortunately, Kaplan–Meier analysis revealed that the individual lncRNAs were not independent prognostic factors (results not shown in this article). However, according to the risk score that was calculated via the five-lncRNA prognostic signature, the samples could be divided into a high-risk group and a low-risk group. In addition, the Kaplan–Meier analysis suggested significant differences in overall survival between the two groups.

Among the five lncRNAs, RP11-128N14.5, RP11-231|13.2, and RP5-894D12.4 are sense intronic lncRNAs; RP11-346L1.2 is a lincRNA, and LAMA5-AS1 is an antisense lncRNA. RP11-128N14.5 has also been detected in non-alcoholic fatty liver disease, and scientists have demonstrated that RP11-128N14.5 is upregulated at the intracellular, extracellular, and histological levels in non-alcoholic fatty liver disease [24]. Shen et al. [25] demonstrated that LAMA5-AS1 is related to the overall survival rate of patients with multiple myeloma. These research findings suggest that RP11-128N14.5 and LAMA5-AS1 are potential prognostic biomarkers for tumours. Our study also demonstrated that these lncRNAs show a close relationship with tumour prognosis.

Although it is well known that immunology regulations may affect cancer development [26], these five lncRNAs associated with osteosarcoma prognosis are rarely studied in immunodeficiency. In addition, the mRNA modifications are potentially new insights into biological basis [27, 28], especially N4-Acetylcytidine on RNA expression [29], these is no studies exploring the role of these five signatures in mRNA modification, especially N4-Acetylcytidine. Therefore, further researches correlated with immunodeficiency and mRNA modification are needed.

Next, to identify the functions of these five lncRNAs, we analyzed their related PCGs to explore the function of these lncRNAs according to the theory that co-expressed genes are more likely to be functionally related [30]. According to GO functional enrichment analysis, the BPs of the five-lncRNA signature mainly involved multicellular organismal processes, the response to stimulus, reproductive processes, localization, cellular processes, and metabolic processes. The KEGG pathway results suggested that olfactory transduction, steroid hormone biosynthesis, neuroactive ligand-receptor interaction, and arachidonic acid metabolism were related to the five-lncRNA signature. The relation of many PCGs with olfactory transduction in the KEGG pathway results was intriguing. Olfactory transduction has been reported in many different kinds of tumours, such as breast cancer [31], thyroid carcinoma [32], and hepatocellular carcinoma [33]. However, the relationship between olfactory transduction and osteosarcoma has not been reported, which provides a new point for osteosarcoma research.

Finally, PPI network was performed to explore the potential biological interaction or co-expression of lncRNA-correlated PCGs [34, 35]. We found RTP1 played an important role in the PPI network, suggesting RTP1 is a potential biomarker that regulates the prognosis of osteosarcoma. To our knowledge, there is rarely study reveal the mechanism of RTP1 in osteosarcoma, which indicated a new target for diagnosis or treatment of osteosarcoma.

Our study still has some limitations. Firstly, we only conducted high-throughput mRNA-seq profiling data analysis; therefore, for deeply understanding the mechanism, it is necessary for further functional verification of the importance of these five lncRNAs in the prognosis of osteosarcoma. Secondly, we only analyzed the data from the TARGET database with limited sample size, therefore other related omic data should be further collected to identify more significant biomarkers that could be interacting with the identified lncRNAs to jointly affect the survival status in a multilayer genetic regulation network [34–36]. Lastly, this study did not explore the possibility to construct a machine-learning tool based on these lncRNAs for prediction of survival due to technical reasons, and we will further try to use the biomarkers to predict the survival based on machine learning tools for better osteosarcoma survival prediction [37, 38].

## Conclusions

In summary, we performed univariate and multivariate Cox regression analyses to identify five lncRNAs related to the survival of patients with osteosarcoma.

## Supplementary Information

**Additional file 1.** Univariate Cox proportional hazards regression analysis of differentially expressed lncRNAs.

**Additional file 2.** Risk score of osteosarcoma patients based on five lncRNAs signature.

**Additional file 3.** Protein-coding genes (PCGs) correlated with five lncRNAs.

## Data Availability

The datasets were downloaded from the TARGET data matrix (https://target-data.nci.nih.gov/Public/OS/mRNA-seq/).

## Abbreviations

LncRNAs: Long noncoding RNAs
TARGET: Therapeutically applicable research to generate effective treatments
ROC: Receiver operating characteristic curve
AUC: Area under the curve
PCGs: Protein-coding genes
mRNA-seq: RNA-sequencing
FC: Fold change
GO: Gene ontology
KEGG: Kyoto encyclopedia of genes and genomes
BP: Biological process
PPI: Protein–protein interaction
