# Supplementary material for: A five metastasis-related long noncoding RNA risk signature for osteosarcoma survival prediction
Source: BMC Med Genomics. 2021 May 8;14:124. doi: 10.1186/s12920-021-00972-5 (PMC8105989; doi:10.1186/s12920-021-00972-5)
Supplement: Supplementary file 1 — Additional file 1. Univariate Cox proportional hazards regression analysis of differentially expressed lncRNAs. [file 12920_2021_972_MOESM1_ESM.docx]

Additional file 1: Table S1 Univariate Cox proportional hazards regression analysis of differentially expressed lncRNAs

| LncRNA | HR | z | pvalue |
| --- | --- | --- | --- |
| RP11-346L1.2 | 1.379241 | 2.896879 | 0.003769 |
| RP5-894D12.4 | 1.470435 | 2.884436 | 0.003921 |
| RP11-804A23.2 | 1.427734 | 2.639999 | 0.008291 |
| RP11-231I13.2 | 1.499193 | 2.593565 | 0.009499 |
| RP11-635O16.2 | 1.416829 | 2.441962 | 0.014608 |
| TUSC7 | 1.466664 | 2.420006 | 0.01552 |
| RP11-128N14.5 | 1.340221 | 2.304819 | 0.021177 |
| RP1-29C18.8 | 1.290329 | 2.143798 | 0.032049 |
| RP11-352D3.2 | 1.257125 | 2.13516 | 0.032748 |
| LAMA5-AS1 | 1.235037 | 2.12525 | 0.033566 |
| CTB-113P19.3 | 0.762333 | -2.01941 | 0.043445 |
| AC091705.1 | 1.438011 | 2.013322 | 0.044081 |
| AC060834.3 | 0.702134 | -1.97914 | 0.0478 |
| RP11-542G1.3 | 1.346654 | 1.914559 | 0.055549 |
| RP1-288H2.2 | 1.302062 | 1.895136 | 0.058074 |
| LL22NC03-86D4.1 | 1.275458 | 1.808669 | 0.070502 |
| RP4-683M8.2 | 1.300162 | 1.728516 | 0.083896 |
| LINC00661 | 1.193593 | 1.59232 | 0.111313 |
| RP11-541P9.3 | 1.259647 | 1.525995 | 0.127011 |
| FEZF1-AS1 | 0.87986 | -1.4881 | 0.136724 |
| RP11-115J16.2 | 1.185154 | 1.484907 | 0.137568 |
| U91328.21 | 0.764361 | -1.46909 | 0.141807 |
| LINC01054 | 1.199609 | 1.42808 | 0.153269 |
| ZNF295-AS1 | 1.182579 | 1.412626 | 0.157766 |
| AC022182.1 | 1.16094 | 1.354006 | 0.175734 |
| LINC00207 | 1.200262 | 1.306223 | 0.191477 |
| RP11-314C16.1 | 0.862113 | -1.2889 | 0.197433 |
| RP1-273G13.3 | 0.871923 | -1.21719 | 0.223531 |
| CTC-551A13.1 | 0.754977 | -1.21612 | 0.223939 |
| CTD-2587H24.5 | 1.152675 | 1.194984 | 0.232093 |
| RP11-572C15.5 | 1.210903 | 1.158514 | 0.246654 |
| LINC00261 | 1.102352 | 1.156327 | 0.247548 |
| CTD-3216D2.5 | 1.190646 | 1.156146 | 0.247622 |
| RP5-1051H14.2 | 1.148676 | 1.063186 | 0.287698 |
| RP11-476H16.1 | 1.187 | 1.052923 | 0.292376 |
| LINC01119 | 0.889973 | -0.9954 | 0.319539 |
| MIR155HG | 1.103333 | 0.926593 | 0.354138 |
| RP11-629E24.2 | 0.916129 | -0.87457 | 0.381808 |
| RP1-38C16.2 | 1.199844 | 0.866647 | 0.386136 |
| RP11-513G11.3 | 0.890921 | -0.84086 | 0.400425 |
| RP11-332K15.1 | 1.115749 | 0.816703 | 0.414098 |
| LINC01183 | 0.88005 | -0.81009 | 0.417886 |
| FZD10-AS1 | 1.133786 | 0.796279 | 0.42587 |
| RP11-259O18.4 | 1.14135 | 0.75973 | 0.447416 |
| RP11-557H15.4 | 1.111662 | 0.75565 | 0.449859 |
| RP1-302G2.5 | 0.936837 | -0.72522 | 0.468316 |
| CTD-2091N23.1 | 0.874852 | -0.72353 | 0.469351 |
| AC008103.3 | 1.186925 | 0.687264 | 0.491916 |
| AC195454.1 | 1.087061 | 0.67085 | 0.502316 |
| AC073626.2 | 1.265322 | 0.630517 | 0.528357 |
| IL21-AS1 | 0.948882 | -0.58638 | 0.557622 |
| RP11-362F19.1 | 1.042676 | 0.51364 | 0.607504 |
| RP1-63M2.5 | 1.06882 | 0.461656 | 0.644328 |
| RP11-1094M14.12 | 1.094228 | 0.450528 | 0.652329 |
| RP11-554D15.3 | 0.949878 | -0.43988 | 0.660021 |
| CTC-527H23.3 | 0.939707 | -0.42019 | 0.674347 |
| LINC00689 | 0.961153 | -0.41496 | 0.678174 |
| RP11-454P21.1 | 0.952657 | -0.38838 | 0.697738 |
| RP11-199F11.2 | 0.96924 | -0.31784 | 0.750606 |
| MIR325HG | 0.966993 | -0.31397 | 0.753541 |
| AP001476.4 | 1.029402 | 0.278502 | 0.780627 |
| RP3-390M24.1 | 1.07652 | 0.255223 | 0.798551 |
| RP11-1082L8.4 | 0.96294 | -0.24432 | 0.806983 |
| CTA-407F11.8 | 0.968081 | -0.24167 | 0.809037 |
| RP11-496H1.2 | 1.022989 | 0.19478 | 0.845565 |
| RP11-493E12.3 | 1.018389 | 0.165008 | 0.868938 |
| RP11-323I1.1 | 0.96728 | -0.15305 | 0.878358 |
| GS1-72M22.1 | 0.987631 | -0.14968 | 0.881017 |
| RP11-431M3.1 | 0.984107 | -0.13693 | 0.891087 |
| RP11-665I14.1 | 1.017158 | 0.125152 | 0.900403 |
| RP1-102D24.5 | 0.989591 | -0.08917 | 0.928945 |
| RP11-136K14.3 | 1.012075 | 0.077751 | 0.938026 |
| RP3-395P12.2 | 0.985772 | -0.06757 | 0.946127 |
| MESTIT1 | 1.008508 | 0.047918 | 0.961782 |
| RP11-20G13.1 | 1.00343 | 0.033157 | 0.973549 |
