# Supplementary material for: A five metastasis-related long noncoding RNA risk signature for osteosarcoma survival prediction
Source: BMC Med Genomics. 2021 May 8;14:124. doi: 10.1186/s12920-021-00972-5 (PMC8105989; doi:10.1186/s12920-021-00972-5)
Supplement: Supplementary file 2 — Additional file 2. Risk score of osteosarcoma patients based on five lncRNAs signature. [file 12920_2021_972_MOESM2_ESM.docx]

Additional file 2: Table S2 Risk score of osteosarcoma patients based on five lncRNAs signature

| id | Survival time | Survival status | Risk Score | Risk level |
| --- | --- | --- | --- | --- |
| TARGET-40-0A4HX8 | 4.794521 | 0 | 0.770115562 | low |
| TARGET-40-0A4I3W | 4.484932 | 1 | 1.834558781 | high |
| TARGET-40-PASRNE | 4.676712 | 0 | 0.562147797 | low |
| TARGET-40-0A4I9K | 6.183562 | 0 | 0.679761033 | low |
| TARGET-40-PAVCLP | 1.4 | 0 | 0.403297801 | low |
| TARGET-40-NAASJI | 1.063014 | 1 | 1.448162637 | high |
| TARGET-40-PANZZJ | 4.213699 | 0 | 0.362514167 | low |
| TARGET-40-PATJVI | 4.315068 | 0 | 0.475622051 | low |
| TARGET-40-PASEBY | 5.613699 | 0 | 1.051824145 | high |
| TARGET-40-PATEEM | 3.975342 | 0 | 1.036929245 | high |
| TARGET-40-0A4I48 | 4.427397 | 0 | 1.761185434 | high |
| TARGET-40-NAAEDH | 2.80274 | 1 | 4.779415117 | high |
| TARGET-40-0A4I5B | 1.69589 | 0 | 0.816822984 | low |
| TARGET-40-PALKDP | 8.991781 | 0 | 1.143824534 | high |
| TARGET-40-PATKSS | 2.076712 | 1 | 0.736272671 | low |
| TARGET-40-PAMYYJ | 0.742466 | 1 | 0.981480026 | low |
| TARGET-40-PAUUML | 1.841096 | 0 | 1.146453539 | high |
| TARGET-40-PAUBIT | 2.819178 | 0 | 0.272902025 | low |
| TARGET-40-NAAEDA | 1.378082 | 1 | 17.09009989 | high |
| TARGET-40-NAAEDD | 1.035616 | 1 | 2.047010262 | high |
| TARGET-40-PAPKWD | 1.156164 | 1 | 0.53896907 | low |
| TARGET-40-0A4I6O | 5.221918 | 1 | 0.418726385 | low |
| TARGET-40-PAUXPZ | 1.660274 | 1 | 3.147530991 | high |
| TARGET-40-PASYUK | 5.123288 | 0 | 1.858387135 | high |
| TARGET-40-PARDAX | 1.860274 | 1 | 0.50553832 | low |
| TARGET-40-PASSLM | 5.109589 | 0 | 1.386191205 | high |
| TARGET-40-PAKFVX | 2.526027 | 0 | 0.904664144 | low |
| TARGET-40-PARFTG | 1.789041 | 1 | 1.305355067 | high |
| TARGET-40-PAKZZK | 4.150685 | 1 | 0.297249518 | low |
| TARGET-40-PANSEN | 6.180822 | 0 | 1.108537894 | high |
| TARGET-40-PANGPE | 9.254795 | 0 | 0.521725938 | low |
| TARGET-40-PASKZZ | 1.484932 | 0 | 0.879861539 | low |
| TARGET-40-PALFYN | 2.747945 | 1 | 0.968462265 | low |
| TARGET-40-PALZGU | 7.89589 | 1 | 1.301929013 | high |
| TARGET-40-PAMEKS | 2.347945 | 1 | 0.524240122 | low |
| TARGET-40-PASEFS | 5.786301 | 0 | 1.354455437 | high |
| TARGET-40-PAVDTY | 1.010959 | 0 | 0.376132522 | low |
| TARGET-40-PAPXGT | 6.920548 | 0 | 0.63874135 | low |
| TARGET-40-PASUUH | 4.452055 | 0 | 0.758256268 | low |
| TARGET-40-0A4I4A | 4.336986 | 0 | 1.22461917 | high |
| TARGET-40-PANVJJ | 8.479452 | 0 | 0.444086642 | low |
| TARGET-40-PAPNVD | 2.526027 | 1 | 3.455713361 | high |
| TARGET-40-PAMLKS | 0.493151 | 1 | 8.697698601 | high |
| TARGET-40-0A4HXS | 8.076712 | 0 | 1.12428074 | high |
| TARGET-40-PANGRW | 9.254795 | 0 | 1.543579577 | high |
| TARGET-40-PARJXU | 4.468493 | 1 | 1.252443911 | high |
| TARGET-40-PAMTCM | 8.224658 | 0 | 0.535401402 | low |
| TARGET-40-PATAWV | 4.663014 | 0 | 1.038872547 | high |
| TARGET-40-PANZHX | 3.624658 | 0 | 0.424713296 | low |
| TARGET-40-PALECC | 2.065753 | 1 | 1.606340665 | high |
| TARGET-40-0A4I65 | 16 | 0 | 0.294659898 | low |
| TARGET-40-0A4I0W | 1.871233 | 0 | 0.511658818 | low |
| TARGET-40-PAPWWC | 6.90411 | 0 | 0.163612912 | low |
| TARGET-40-PAMJXS | 10.58082 | 1 | 1.2270675 | high |
| TARGET-40-PALKGN | 5.813699 | 0 | 0.207144702 | low |
| TARGET-40-0A4I0Q | 1.421918 | 1 | 0.823174717 | low |
| TARGET-40-PAVALD | 1.509589 | 0 | 0.784006792 | low |
| TARGET-40-PANMIG | 2.126027 | 1 | 1.810710106 | high |
| TARGET-40-0A4HY5 | 0.794521 | 1 | 2.587469304 | high |
| TARGET-40-PARGTM | 7.150685 | 0 | 0.60540531 | low |
| TARGET-40-PAKXLD | 6.745205 | 0 | 0.325747784 | low |
| TARGET-40-0A4I8S | 2.158904 | 1 | 1.780018226 | high |
| TARGET-40-0A4HLD | 10.81096 | 0 | 2.740002285 | high |
| TARGET-40-PARKAF | 2.906849 | 1 | 0.430026292 | low |
| TARGET-40-PAMRHD | 0.20274 | 1 | 1.557533777 | high |
| TARGET-40-0A4I3S | 5.052055 | 0 | 0.990718057 | high |
| TARGET-40-0A4HMC | 1.032877 | 0 | 0.670564656 | low |
| TARGET-40-PATMPU | 4.326027 | 1 | 0.980582916 | low |
| TARGET-40-PATPBS | 4.021918 | 0 | 0.660569683 | low |
| TARGET-40-PAUYTT | 1.586301 | 0 | 0.8917447 | low |
| TARGET-40-0A4I8U | 2.693151 | 0 | 0.996341876 | high |
| TARGET-40-0A4I4O | 1.717808 | 1 | 3.054329458 | high |
| TARGET-40-PAUTWB | 1.882192 | 0 | 0.38650433 | low |
| TARGET-40-PAPIJR | 4.709589 | 0 | 0.511106859 | low |
| TARGET-40-PAUVUL | 0.950685 | 1 | 16.1031255 | high |
| TARGET-40-NAASJK | 2.071233 | 1 | 2.711047451 | high |
| TARGET-40-PASFCV | 5.649315 | 0 | 0.468449791 | low |
| TARGET-40-0A4I8O | 2.490411 | 1 | 1.9967562 | high |
| TARGET-40-PASNZV | 4.786301 | 0 | 0.783298194 | low |
| TARGET-40-0A4I4M | 1.939726 | 0 | 2.845280345 | high |
| TARGET-40-PAVECB | 1.30411 | 0 | 0.474552375 | low |
| TARGET-40-PANXSC | 3.334247 | 0 | 1.161029212 | high |
| TARGET-40-PATMIF | 4.328767 | 0 | 0.753778643 | low |
| TARGET-40-PAUTYB | 1.471233 | 1 | 0.66775151 | low |
| TARGET-40-PAKUZU | 0 | 0 | 0.502072472 | low |
| TARGET-40-PALHRL | 11.98356 | 0 | 0.890838123 | low |
| TARGET-40-PATUXZ | 1.39726 | 1 | 2.358136897 | high |
| TARGET-40-0A4I42 | 1.69589 | 1 | 1.668303813 | high |
| TARGET-40-PARBGW | 0.783562 | 0 | 2.084179204 | high |
| TARGET-40-PAMHYN | 10.68493 | 0 | 1.615124371 | high |
| TARGET-40-PATMXR | 1.057534 | 1 | 2.389445895 | high |
| TARGET-40-NAAEDI | 0.989041 | 1 | 1.079436097 | high |
| TARGET-40-PAMHLF | 5.241096 | 0 | 0.60068693 | low |
| TARGET-40-PANPUM | 0.808219 | 1 | 1.147922047 | high |
| TARGET-40-PALWWX | 5.926027 | 0 | 0.996127061 | high |
