# Supplementary material for: A five metastasis-related long noncoding RNA risk signature for osteosarcoma survival prediction
Source: BMC Med Genomics. 2021 May 8;14:124. doi: 10.1186/s12920-021-00972-5 (PMC8105989; doi:10.1186/s12920-021-00972-5)
Supplement: Supplementary file 3 — Additional file 3. Protein-coding genes (PCGs) correlated with five lncRNAs. [file 12920_2021_972_MOESM3_ESM.docx]

Additional file 3: Table S3 Protein-coding genes (PCGs) correlated with five lncRNAs

| PCGs | Pearson coefficient | Pvalue |
| --- | --- | --- |
| LAMA5 | 0.543 | 7.57E-09 |
| KHDC3L | 0.599 | 7.46E-11 |
| SORCS3 | 0.562 | 1.71E-09 |
| RNF183 | 0.529 | 2.17E-08 |
| CRH | 0.523 | 3.36E-08 |
| CALML5 | 0.52 | 3.98E-08 |
| GAL3ST1 | 0.507 | 9.99E-08 |
| CFAP126 | 0.507 | 9.95E-08 |
| PRR5-ARHGAP8 | 0.501 | 1.47E-07 |
| KRTAP19-6 | 0.665 | 8.15E-14 |
| MUC17 | 0.53 | 1.99E-08 |
| HSD17B13 | 0.516 | 5.42E-08 |
| OR10G2 | 0.51 | 8.27E-08 |
| TRIM49C | 0.502 | 1.41E-07 |
| C9 | 0.502 | 1.40E-07 |
| DPPA3 | 0.586 | 2.38E-10 |
| AGXT2 | 0.584 | 2.78E-10 |
| OR4F5 | 0.572 | 7.81E-10 |
| SLC17A4 | 0.562 | 1.76E-09 |
| BEND2 | 0.556 | 2.73E-09 |
| GALNTL5 | 0.543 | 7.62E-09 |
| DEFB125 | 0.528 | 2.37E-08 |
| NUTM1 | 0.524 | 3.12E-08 |
| OR2A12 | 0.521 | 3.90E-08 |
| OCM2 | 0.521 | 3.76E-08 |
| PTCHD4 | 0.518 | 4.62E-08 |
| HTN1 | 0.513 | 6.80E-08 |
| CFTR | 0.513 | 6.41E-08 |
| CABS1 | 0.513 | 6.59E-08 |
| ATP10B | 0.51 | 8.28E-08 |
| DYTN | 0.508 | 9.27E-08 |
| DMRTC2 | 0.506 | 1.09E-07 |
| SLC17A3 | 0.505 | 1.15E-07 |
| OR56A4 | 0.505 | 1.18E-07 |
| OR4C13 | 0.503 | 1.31E-07 |
| KRT20 | 0.501 | 1.53E-07 |
| SULT1E1 | 0.5 | 1.54E-07 |
| CASP14 | 0.766 | 4.00E-20 |
| OR7D4 | 0.747 | 1.06E-18 |
| CYP4F3 | 0.747 | 1.06E-18 |
| LRRC74B | 0.745 | 1.47E-18 |
| PRR27 | 0.742 | 2.42E-18 |
| CYP1A2 | 0.739 | 3.71E-18 |
| CCL16 | 0.737 | 5.30E-18 |
| MTRNR2L4 | 0.736 | 5.96E-18 |
| KLB | 0.731 | 1.33E-17 |
| TMEM213 | 0.731 | 1.21E-17 |
| OR4D9 | 0.73 | 1.56E-17 |
| GFI1B | 0.729 | 1.65E-17 |
| CFAP69 | 0.726 | 2.54E-17 |
| ATCAY | 0.725 | 3.35E-17 |
| NANOG | 0.722 | 5.24E-17 |
| LECT2 | 0.721 | 5.40E-17 |
| IQCF3 | 0.719 | 7.66E-17 |
| FCAR | 0.718 | 8.35E-17 |
| MOGAT3 | 0.717 | 1.02E-16 |
| C2orf83 | 0.715 | 1.37E-16 |
| OR4E2 | 0.714 | 1.51E-16 |
| TMEM78 | 0.712 | 2.16E-16 |
| TRIM73 | 0.71 | 2.87E-16 |
| CEACAM8 | 0.709 | 3.00E-16 |
| OR1M1 | 0.707 | 4.36E-16 |
| TMPRSS4 | 0.707 | 4.11E-16 |
| OR2A5 | 0.706 | 4.96E-16 |
| SLC7A14 | 0.705 | 5.47E-16 |
| ATP5L2 | 0.703 | 7.37E-16 |
| C1orf64 | 0.703 | 7.37E-16 |
| PLCXD3 | 0.703 | 6.83E-16 |
| NCMAP | 0.702 | 7.56E-16 |
| RTP1 | 0.702 | 8.30E-16 |
| SVOP | 0.702 | 8.37E-16 |
| CYP4F8 | 0.701 | 8.96E-16 |
| OR7A17 | 0.7 | 1.11E-15 |
| FAM153B | 0.7 | 1.01E-15 |
| OR52I1 | 0.699 | 1.15E-15 |
| OR10G3 | 0.699 | 1.21E-15 |
| FADS6 | 0.697 | 1.65E-15 |
| RP11-310N16.1 | 0.693 | 2.70E-15 |
| MAS1 | 0.693 | 2.56E-15 |
| TISP43 | 0.691 | 3.61E-15 |
| ACSM2A | 0.691 | 3.39E-15 |
| SH2D3A | 0.69 | 3.89E-15 |
| TPH2 | 0.69 | 3.85E-15 |
| FUT6 | 0.689 | 4.30E-15 |
| LRRD1 | 0.688 | 4.97E-15 |
| DYNAP | 0.688 | 5.34E-15 |
| POU5F2 | 0.687 | 5.80E-15 |
| OR7G2 | 0.687 | 5.74E-15 |
| EREG | 0.687 | 6.03E-15 |
| HRH4 | 0.686 | 6.10E-15 |
| ADGRD2 | 0.686 | 6.41E-15 |
| OR2V2 | 0.686 | 6.64E-15 |
| LIN28A | 0.685 | 7.28E-15 |
| COX6B2 | 0.685 | 7.42E-15 |
| RASSF6 | 0.684 | 8.71E-15 |
| EXD1 | 0.684 | 8.10E-15 |
| FABP2 | 0.684 | 8.39E-15 |
| TSPAN19 | 0.684 | 8.18E-15 |
| IYD | 0.683 | 9.38E-15 |
| CRX | 0.683 | 9.35E-15 |
| FAM179A | 0.683 | 8.94E-15 |
| AKR1D1 | 0.682 | 1.01E-14 |
| OR6Y1 | 0.682 | 1.12E-14 |
| RD3 | 0.681 | 1.14E-14 |
| OR11G2 | 0.681 | 1.23E-14 |
| NUGGC | 0.681 | 1.22E-14 |
| KCTD16 | 0.68 | 1.43E-14 |
| PLEKHD1 | 0.68 | 1.37E-14 |
| RGSL1 | 0.68 | 1.44E-14 |
| CNTN2 | 0.68 | 1.42E-14 |
| TAT | 0.679 | 1.61E-14 |
| OR51M1 | 0.678 | 1.65E-14 |
| PIGR | 0.678 | 1.71E-14 |
| DPPA2 | 0.675 | 2.42E-14 |
| OR1D2 | 0.675 | 2.60E-14 |
| VWA5B1 | 0.672 | 3.41E-14 |
| PLA2G4D | 0.672 | 3.46E-14 |
| USP26 | 0.672 | 3.56E-14 |
| ADGRF1 | 0.671 | 4.02E-14 |
| GGT6 | 0.671 | 4.24E-14 |
| KLK11 | 0.671 | 4.08E-14 |
| RP11-437B10.1 | 0.67 | 4.32E-14 |
| FAM83A | 0.67 | 4.50E-14 |
| KIAA1257 | 0.67 | 4.74E-14 |
| OR10H1 | 0.67 | 4.50E-14 |
| SLC35G3 | 0.669 | 5.34E-14 |
| OR8D4 | 0.669 | 5.24E-14 |
| ACSL6 | 0.669 | 5.06E-14 |
| SYT5 | 0.669 | 5.22E-14 |
| OR10A2 | 0.669 | 5.00E-14 |
| OR2B11 | 0.668 | 5.81E-14 |
| OR4K13 | 0.668 | 5.44E-14 |
| AL357140.1 | 0.667 | 6.19E-14 |
| OR5H14 | 0.667 | 6.81E-14 |
| SLC7A9 | 0.667 | 6.29E-14 |
| AL023806.1 | 0.667 | 6.36E-14 |
| BCL2L15 | 0.666 | 7.28E-14 |
| PPP5D1 | 0.666 | 7.01E-14 |
| ERN2 | 0.666 | 7.49E-14 |
| C17orf105 | 0.665 | 7.72E-14 |
| PADI4 | 0.665 | 8.43E-14 |
| FCRL5 | 0.665 | 8.11E-14 |
| BCL2L14 | 0.664 | 9.41E-14 |
| RGR | 0.663 | 1.00E-13 |
| GPX2 | 0.663 | 9.89E-14 |
| SPATA6L | 0.663 | 1.05E-13 |
| ADH4 | 0.662 | 1.19E-13 |
| CDC20B | 0.662 | 1.09E-13 |
| C2orf81 | 0.662 | 1.18E-13 |
| PPEF2 | 0.661 | 1.33E-13 |
| GRK7 | 0.661 | 1.33E-13 |
| TRAPPC3L | 0.661 | 1.30E-13 |
| MCF2L2 | 0.66 | 1.38E-13 |
| MOG | 0.66 | 1.45E-13 |
| OPRM1 | 0.66 | 1.50E-13 |
| CTRC | 0.66 | 1.49E-13 |
| RAX2 | 0.659 | 1.66E-13 |
| C8orf86 | 0.659 | 1.60E-13 |
| OR5AU1 | 0.658 | 1.82E-13 |
| BSND | 0.658 | 1.77E-13 |
| CLEC17A | 0.658 | 1.74E-13 |
| IZUMO1 | 0.658 | 1.81E-13 |
| MSANTD1 | 0.658 | 1.74E-13 |
| SAG | 0.658 | 1.76E-13 |
| TM4SF20 | 0.657 | 2.11E-13 |
| CNGB1 | 0.656 | 2.38E-13 |
| FLG2 | 0.656 | 2.32E-13 |
| SLC13A1 | 0.656 | 2.19E-13 |
| KIR3DX1 | 0.655 | 2.40E-13 |
| ADAM32 | 0.654 | 2.70E-13 |
| TMC2 | 0.653 | 3.34E-13 |
| AL356289.1 | 0.653 | 3.01E-13 |
| WNT9B | 0.653 | 3.15E-13 |
| NPAP1 | 0.652 | 3.48E-13 |
| NXNL2 | 0.652 | 3.50E-13 |
| ADRB3 | 0.652 | 3.60E-13 |
| OR2M4 | 0.652 | 3.43E-13 |
| TTPA | 0.651 | 4.06E-13 |
| CD300LD | 0.651 | 3.90E-13 |
| EYS | 0.65 | 4.28E-13 |
| PDE6A | 0.65 | 4.32E-13 |
| TSGA13 | 0.65 | 4.40E-13 |
| PDE6C | 0.65 | 4.54E-13 |
| CHST9 | 0.65 | 4.57E-13 |
| BPIFB1 | 0.649 | 5.04E-13 |
| FAM129C | 0.649 | 4.89E-13 |
| CYP2B6 | 0.648 | 5.41E-13 |
| AIPL1 | 0.648 | 5.30E-13 |
| PLIN5 | 0.648 | 5.34E-13 |
| CELA2A | 0.648 | 5.57E-13 |
| RPH3A | 0.648 | 5.76E-13 |
| SULT2A1 | 0.647 | 5.87E-13 |
| SLC26A8 | 0.646 | 7.07E-13 |
| SEC14L3 | 0.646 | 6.58E-13 |
| RIMS2 | 0.646 | 6.99E-13 |
| IFNW1 | 0.646 | 6.85E-13 |
| GCNT3 | 0.645 | 7.30E-13 |
| OR4K2 | 0.645 | 7.58E-13 |
| PCDH15 | 0.645 | 7.31E-13 |
| TSPAN16 | 0.644 | 8.19E-13 |
| ARGFX | 0.644 | 8.44E-13 |
| OR10J1 | 0.644 | 8.80E-13 |
| MROH7 | 0.644 | 8.82E-13 |
| ERVW-1 | 0.644 | 8.25E-13 |
| MYLK3 | 0.643 | 9.34E-13 |
| SLFN12L | 0.642 | 1.03E-12 |
| TMEM239 | 0.642 | 1.01E-12 |
| RHBG | 0.642 | 1.01E-12 |
| OR1I1 | 0.641 | 1.11E-12 |
| GP2 | 0.641 | 1.20E-12 |
| PROM2 | 0.64 | 1.36E-12 |
| OR2G6 | 0.64 | 1.36E-12 |
| NANOGNB | 0.64 | 1.29E-12 |
| FCRL2 | 0.64 | 1.35E-12 |
| CATSPERB | 0.639 | 1.37E-12 |
| PDILT | 0.639 | 1.41E-12 |
| PATE2 | 0.639 | 1.38E-12 |
| PCDHA9 | 0.639 | 1.38E-12 |
| PKHD1L1 | 0.639 | 1.42E-12 |
| OR1C1 | 0.639 | 1.41E-12 |
| CCDC79 | 0.639 | 1.45E-12 |
| CATIP | 0.638 | 1.54E-12 |
| NWD1 | 0.638 | 1.54E-12 |
| FAM221B | 0.638 | 1.58E-12 |
| CHRNB4 | 0.638 | 1.64E-12 |
| C12orf50 | 0.637 | 1.71E-12 |
| OR5H1 | 0.637 | 1.82E-12 |
| PPP1R42 | 0.637 | 1.80E-12 |
| TTC22 | 0.637 | 1.78E-12 |
| CASR | 0.637 | 1.86E-12 |
| LY6G6E | 0.637 | 1.82E-12 |
| GLRA1 | 0.636 | 2.04E-12 |
| TMEM236 | 0.636 | 1.99E-12 |
| ART4 | 0.636 | 1.97E-12 |
| FAM153C | 0.636 | 1.89E-12 |
| AC087762.1 | 0.635 | 2.26E-12 |
| C10orf82 | 0.635 | 2.19E-12 |
| KCNA7 | 0.635 | 2.25E-12 |
| MAP3K15 | 0.635 | 2.24E-12 |
| ZNF645 | 0.634 | 2.42E-12 |
| PRSS27 | 0.634 | 2.36E-12 |
| NLRP12 | 0.634 | 2.40E-12 |
| LRRC69 | 0.634 | 2.41E-12 |
| OR2A25 | 0.633 | 2.57E-12 |
| TTC16 | 0.632 | 2.83E-12 |
| OR51D1 | 0.632 | 2.97E-12 |
| CATSPERD | 0.632 | 2.97E-12 |
| LDHAL6A | 0.631 | 3.15E-12 |
| PIH1D3 | 0.631 | 3.15E-12 |
| TPH1 | 0.631 | 3.28E-12 |
| INMT | 0.631 | 3.43E-12 |
| C6orf141 | 0.63 | 3.65E-12 |
| OR6C75 | 0.63 | 3.60E-12 |
| DYDC1 | 0.629 | 4.20E-12 |
| FFAR1 | 0.629 | 3.88E-12 |
| C10orf53 | 0.629 | 4.03E-12 |
| NR2E3 | 0.629 | 3.88E-12 |
| LGSN | 0.629 | 3.95E-12 |
| TEPP | 0.628 | 4.56E-12 |
| SLC1A6 | 0.628 | 4.36E-12 |
| NOTO | 0.627 | 5.17E-12 |
| CCDC169 | 0.627 | 4.91E-12 |
| OR4D1 | 0.627 | 4.76E-12 |
| RNF222 | 0.627 | 4.83E-12 |
| CCDC30 | 0.626 | 5.37E-12 |
| DOC2A | 0.626 | 5.47E-12 |
| KCNG4 | 0.626 | 5.69E-12 |
| SERPINB10 | 0.626 | 5.49E-12 |
| AC007040.11 | 0.625 | 6.04E-12 |
| ALDH8A1 | 0.625 | 6.24E-12 |
| OR4P4 | 0.625 | 6.25E-12 |
| CA6 | 0.624 | 6.62E-12 |
| CSTL1 | 0.624 | 6.81E-12 |
| GBP6 | 0.623 | 7.21E-12 |
| HAMP | 0.623 | 7.50E-12 |
| OR2AT4 | 0.623 | 7.64E-12 |
| TMEM156 | 0.623 | 7.07E-12 |
| OR11A1 | 0.622 | 8.21E-12 |
| ANKRD30A | 0.622 | 8.49E-12 |
| C8orf74 | 0.621 | 9.42E-12 |
| OR4K17 | 0.621 | 9.08E-12 |
| TNS4 | 0.621 | 9.34E-12 |
| ATP1A4 | 0.621 | 9.01E-12 |
| FCRL1 | 0.621 | 9.39E-12 |
| PRR23C | 0.621 | 8.65E-12 |
| C1QTNF9 | 0.62 | 9.86E-12 |
| ECT2L | 0.62 | 1.03E-11 |
| CDH9 | 0.62 | 1.03E-11 |
| CFAP54 | 0.62 | 9.54E-12 |
| WFDC11 | 0.619 | 1.09E-11 |
| AMER2 | 0.619 | 1.10E-11 |
| KLF17 | 0.619 | 1.14E-11 |
| PNLIPRP1 | 0.618 | 1.25E-11 |
| A2ML1 | 0.618 | 1.18E-11 |
| MOBP | 0.618 | 1.16E-11 |
| VSIG1 | 0.617 | 1.37E-11 |
| CATSPERG | 0.617 | 1.31E-11 |
| MDS2 | 0.616 | 1.44E-11 |
| OR14J1 | 0.616 | 1.52E-11 |
| OR5AS1 | 0.616 | 1.44E-11 |
| FAM71F2 | 0.616 | 1.51E-11 |
| CCDC81 | 0.616 | 1.42E-11 |
| ADAMTSL3 | 0.616 | 1.46E-11 |
| TPRXL | 0.615 | 1.64E-11 |
| SLC19A3 | 0.615 | 1.68E-11 |
| SLC36A3 | 0.614 | 1.76E-11 |
| HSD17B2 | 0.614 | 1.82E-11 |
| FAM92B | 0.614 | 1.84E-11 |
| IDO2 | 0.613 | 1.92E-11 |
| LDLRAD1 | 0.613 | 2.04E-11 |
| DDX25 | 0.613 | 1.96E-11 |
| PLD5 | 0.613 | 1.95E-11 |
| SLCO1A2 | 0.612 | 2.23E-11 |
| ADGRF2 | 0.612 | 2.21E-11 |
| AGXT | 0.612 | 2.26E-11 |
| OR1A1 | 0.612 | 2.14E-11 |
| G6PC | 0.612 | 2.20E-11 |
| OR8G1 | 0.611 | 2.46E-11 |
| TMCO2 | 0.61 | 2.70E-11 |
| ANKRD62 | 0.61 | 2.53E-11 |
| PRSS37 | 0.61 | 2.72E-11 |
| ASB15 | 0.61 | 2.66E-11 |
| PRDM7 | 0.61 | 2.66E-11 |
| LY9 | 0.61 | 2.53E-11 |
| C17orf99 | 0.609 | 2.90E-11 |
| ASB18 | 0.609 | 2.89E-11 |
| MEIOC | 0.609 | 2.93E-11 |
| RBFOX3 | 0.608 | 3.03E-11 |
| CFHR5 | 0.608 | 3.04E-11 |
| GLB1L3 | 0.608 | 3.12E-11 |
| TFAP2D | 0.607 | 3.44E-11 |
| CRLF2 | 0.607 | 3.44E-11 |
| PLA2G2C | 0.606 | 3.77E-11 |
| SLC12A3 | 0.606 | 3.68E-11 |
| IGFL4 | 0.606 | 3.95E-11 |
| LHFPL5 | 0.605 | 4.32E-11 |
| GABRA1 | 0.605 | 4.09E-11 |
| MRLN | 0.605 | 4.28E-11 |
| OR5A1 | 0.605 | 4.32E-11 |
| BCO2 | 0.605 | 4.34E-11 |
| UGT1A1 | 0.605 | 4.23E-11 |
| GRHL2 | 0.604 | 4.69E-11 |
| CDH1 | 0.604 | 4.49E-11 |
| SLC30A10 | 0.603 | 4.91E-11 |
| PRSS38 | 0.603 | 4.95E-11 |
| SLC5A8 | 0.603 | 5.03E-11 |
| AC004076.7 | 0.603 | 5.14E-11 |
| C12orf42 | 0.603 | 4.97E-11 |
| SCGB2A2 | 0.603 | 5.14E-11 |
| LINC01620 | 0.603 | 5.21E-11 |
| IL12B | 0.603 | 4.90E-11 |
| OR6N1 | 0.602 | 5.49E-11 |
| LCN15 | 0.602 | 5.34E-11 |
| TMEM40 | 0.601 | 6.07E-11 |
| KRTAP9-6 | 0.601 | 5.80E-11 |
| FAM153A | 0.601 | 5.96E-11 |
| DAND5 | 0.601 | 6.21E-11 |
| DPPA4 | 0.6 | 6.43E-11 |
| TMPRSS12 | 0.6 | 6.74E-11 |
| OR2T12 | 0.6 | 6.87E-11 |
| GABRG2 | 0.6 | 6.53E-11 |
| ABCG5 | 0.6 | 6.71E-11 |
| CDHR4 | 0.6 | 6.70E-11 |
| POTEB | 0.6 | 6.85E-11 |
| ESRRG | 0.6 | 6.58E-11 |
| OR10K1 | 0.6 | 6.51E-11 |
| OR2T33 | 0.6 | 6.58E-11 |
| FAM47E | 0.599 | 7.46E-11 |
| KPNA5 | 0.599 | 7.29E-11 |
| SCN7A | 0.599 | 7.03E-11 |
| PLA2G2D | 0.598 | 8.11E-11 |
| ZNF483 | 0.598 | 7.82E-11 |
| CA5A | 0.598 | 7.60E-11 |
| OR2M3 | 0.598 | 7.75E-11 |
| FANCD2OS | 0.598 | 7.77E-11 |
| PRORY | 0.598 | 7.72E-11 |
| BRDT | 0.598 | 7.62E-11 |
| PGBD2 | 0.597 | 9.00E-11 |
| CLEC4C | 0.597 | 8.70E-11 |
| OR7D2 | 0.597 | 8.59E-11 |
| KCNV1 | 0.597 | 9.06E-11 |
| OR7G3 | 0.597 | 9.01E-11 |
| GRM7 | 0.597 | 8.93E-11 |
| CBWD3 | 0.597 | 8.45E-11 |
| DNAJC5G | 0.597 | 8.38E-11 |
| CD226 | 0.596 | 9.30E-11 |
| ANKRD30B | 0.596 | 9.47E-11 |
| TARM1 | 0.596 | 9.55E-11 |
| ZIM3 | 0.596 | 9.51E-11 |
| ACP7 | 0.596 | 9.21E-11 |
| HEPACAM | 0.596 | 9.31E-11 |
| NME9 | 0.596 | 9.45E-11 |
| CLDN16 | 0.596 | 9.90E-11 |
| PRAM1 | 0.595 | 1.07E-10 |
| ONECUT3 | 0.595 | 1.01E-10 |
| PCDHGA8 | 0.595 | 1.05E-10 |
| ELF5 | 0.595 | 1.02E-10 |
| OR8B8 | 0.594 | 1.18E-10 |
| STAR | 0.594 | 1.10E-10 |
| DEFB107B | 0.594 | 1.13E-10 |
| CEACAM20 | 0.594 | 1.18E-10 |
| NLRP7 | 0.594 | 1.10E-10 |
| ZNF157 | 0.594 | 1.19E-10 |
| CYLC2 | 0.594 | 1.16E-10 |
| APOA2 | 0.593 | 1.25E-10 |
| SNTG1 | 0.592 | 1.35E-10 |
| KLRD1 | 0.592 | 1.33E-10 |
| OR56A1 | 0.592 | 1.42E-10 |
| TMEM212 | 0.592 | 1.31E-10 |
| OLAH | 0.592 | 1.38E-10 |
| SLC22A25 | 0.591 | 1.51E-10 |
| SNX31 | 0.591 | 1.48E-10 |
| PRAMEF10 | 0.591 | 1.46E-10 |
| EFCAB12 | 0.591 | 1.52E-10 |
| PRDM14 | 0.591 | 1.51E-10 |
| C2orf50 | 0.59 | 1.66E-10 |
| SLC35G5 | 0.59 | 1.67E-10 |
| OR6M1 | 0.59 | 1.62E-10 |
| CHRM5 | 0.59 | 1.70E-10 |
| ANKRD30BL | 0.589 | 1.74E-10 |
| GAD2 | 0.589 | 1.72E-10 |
| MLXIPL | 0.588 | 1.89E-10 |
| OR2AG2 | 0.588 | 1.99E-10 |
| MROH2B | 0.588 | 2.02E-10 |
| RP11-33O4.2 | 0.588 | 1.98E-10 |
| HTR3B | 0.588 | 1.92E-10 |
| DKK4 | 0.588 | 2.01E-10 |
| PSG5 | 0.588 | 1.90E-10 |
| SLC5A12 | 0.588 | 1.90E-10 |
| MC2R | 0.588 | 1.96E-10 |
| PGLYRP4 | 0.587 | 2.13E-10 |
| OR10K2 | 0.587 | 2.19E-10 |
| FAM159A | 0.587 | 2.08E-10 |
| STRC | 0.587 | 2.13E-10 |
| KCNJ6 | 0.586 | 2.23E-10 |
| FBXO15 | 0.586 | 2.26E-10 |
| PTK6 | 0.586 | 2.37E-10 |
| C6orf25 | 0.585 | 2.64E-10 |
| OR6K3 | 0.585 | 2.55E-10 |
| GOLGA6L2 | 0.585 | 2.54E-10 |
| NRSN1 | 0.585 | 2.63E-10 |
| HCN1 | 0.584 | 2.73E-10 |
| POU5F1B | 0.584 | 2.70E-10 |
| PATE4 | 0.584 | 2.77E-10 |
| OR8H1 | 0.584 | 2.74E-10 |
| TTC34 | 0.584 | 2.78E-10 |
| ELF3 | 0.584 | 2.64E-10 |
| TDGF1 | 0.584 | 2.68E-10 |
| F13B | 0.584 | 2.69E-10 |
| SLC1A2 | 0.584 | 2.67E-10 |
| SPATA3 | 0.584 | 2.67E-10 |
| FAM186A | 0.584 | 2.80E-10 |
| DEFB123 | 0.584 | 2.82E-10 |
| G6PC2 | 0.583 | 3.10E-10 |
| USP29 | 0.583 | 2.96E-10 |
| CLEC19A | 0.583 | 3.11E-10 |
| NLRP8 | 0.583 | 3.07E-10 |
| NAT16 | 0.582 | 3.19E-10 |
| ITIH1 | 0.582 | 3.25E-10 |
| RP11-507M3.1 | 0.582 | 3.23E-10 |
| SLC7A13 | 0.582 | 3.35E-10 |
| OR5A2 | 0.582 | 3.35E-10 |
| SCN1A | 0.582 | 3.33E-10 |
| LPA | 0.581 | 3.44E-10 |
| AXDND1 | 0.581 | 3.71E-10 |
| CST8 | 0.58 | 3.81E-10 |
| LA16c-431H6.6 | 0.58 | 3.81E-10 |
| SIAH3 | 0.58 | 3.76E-10 |
| SPAG11A | 0.58 | 4.04E-10 |
| AADACL3 | 0.58 | 3.76E-10 |
| WDR93 | 0.58 | 3.95E-10 |
| DRD5 | 0.579 | 4.38E-10 |
| SPATA21 | 0.579 | 4.15E-10 |
| CRYGA | 0.579 | 4.28E-10 |
| ACSM2B | 0.579 | 4.07E-10 |
| OFCC1 | 0.578 | 4.80E-10 |
| KCNJ16 | 0.578 | 4.55E-10 |
| SLC13A4 | 0.578 | 4.46E-10 |
| DNAJB7 | 0.578 | 4.67E-10 |
| PKHD1 | 0.578 | 4.54E-10 |
| SLC26A5 | 0.578 | 4.63E-10 |
| PGA3 | 0.578 | 4.70E-10 |
| CYP3A43 | 0.578 | 4.66E-10 |
| C14orf105 | 0.578 | 4.49E-10 |
| TGM5 | 0.578 | 4.77E-10 |
| OR2T2 | 0.578 | 4.65E-10 |
| KRT40 | 0.577 | 4.86E-10 |
| SLC5A5 | 0.577 | 5.12E-10 |
| GPR37L1 | 0.577 | 5.10E-10 |
| LIPH | 0.577 | 4.92E-10 |
| KRT13 | 0.577 | 4.95E-10 |
| SLC26A3 | 0.577 | 5.15E-10 |
| P2RX3 | 0.577 | 4.95E-10 |
| MGAT4C | 0.577 | 4.83E-10 |
| UPB1 | 0.577 | 5.18E-10 |
| CNGA1 | 0.577 | 4.99E-10 |
| POM121L7 | 0.577 | 5.23E-10 |
| MACC1 | 0.576 | 5.41E-10 |
| ESRP2 | 0.576 | 5.36E-10 |
| ACBD7 | 0.576 | 5.33E-10 |
| SLC28A2 | 0.576 | 5.37E-10 |
| PBOV1 | 0.576 | 5.37E-10 |
| SEC14L6 | 0.576 | 5.36E-10 |
| SLC22A11 | 0.575 | 5.93E-10 |
| SLC9A4 | 0.575 | 5.91E-10 |
| WDR66 | 0.575 | 6.01E-10 |
| PDZD7 | 0.575 | 5.83E-10 |
| CNTNAP3 | 0.575 | 5.80E-10 |
| CFHR1 | 0.575 | 6.01E-10 |
| CA14 | 0.575 | 5.82E-10 |
| SNTN | 0.575 | 5.76E-10 |
| STMND1 | 0.575 | 6.16E-10 |
| OR5AN1 | 0.575 | 6.12E-10 |
| ST8SIA5 | 0.574 | 6.28E-10 |
| C1orf168 | 0.574 | 6.60E-10 |
| OR8A1 | 0.574 | 6.29E-10 |
| RASAL1 | 0.573 | 6.81E-10 |
| HCAR3 | 0.573 | 7.27E-10 |
| OR6C4 | 0.573 | 7.08E-10 |
| C2orf91 | 0.573 | 7.27E-10 |
| FBXW12 | 0.573 | 7.04E-10 |
| CCDC27 | 0.572 | 7.57E-10 |
| ACSM6 | 0.572 | 7.70E-10 |
| CYP4F11 | 0.572 | 7.65E-10 |
| TOPAZ1 | 0.572 | 7.67E-10 |
| DAOA | 0.572 | 7.87E-10 |
| OTC | 0.572 | 7.88E-10 |
| ART3 | 0.572 | 7.94E-10 |
| A1CF | 0.572 | 7.35E-10 |
| ACOXL | 0.572 | 7.73E-10 |
| ZNF99 | 0.572 | 7.76E-10 |
| LYZL6 | 0.571 | 8.51E-10 |
| OTUD6A | 0.571 | 8.12E-10 |
| KCTD19 | 0.571 | 8.44E-10 |
| AGBL2 | 0.571 | 7.98E-10 |
| UPK1A | 0.57 | 9.01E-10 |
| PDE4C | 0.57 | 9.08E-10 |
| OR52K1 | 0.57 | 9.02E-10 |
| CFAP221 | 0.57 | 9.23E-10 |
| KCNH6 | 0.57 | 9.21E-10 |
| BEST3 | 0.569 | 9.55E-10 |
| AKR1C4 | 0.569 | 9.42E-10 |
| ZNF716 | 0.569 | 1.00E-09 |
| UQCRHL | 0.569 | 9.43E-10 |
| PCDH11Y | 0.569 | 9.87E-10 |
| VRTN | 0.569 | 9.68E-10 |
| POU2F3 | 0.569 | 9.73E-10 |
| SEC14L5 | 0.569 | 9.93E-10 |
| OR1L8 | 0.568 | 1.10E-09 |
| CCDC38 | 0.568 | 1.08E-09 |
| ZPBP2 | 0.567 | 1.15E-09 |
| OR2V1 | 0.567 | 1.12E-09 |
| DPP10 | 0.567 | 1.11E-09 |
| SDR42E2 | 0.567 | 1.18E-09 |
| OR7A5 | 0.567 | 1.15E-09 |
| FRRS1L | 0.567 | 1.17E-09 |
| CYP4B1 | 0.567 | 1.13E-09 |
| ANKRD34C | 0.567 | 1.11E-09 |
| C2orf15 | 0.566 | 1.30E-09 |
| CAPN8 | 0.566 | 1.25E-09 |
| TCF23 | 0.566 | 1.29E-09 |
| SAMD7 | 0.566 | 1.22E-09 |
| B4GALNT2 | 0.566 | 1.22E-09 |
| SLC5A11 | 0.566 | 1.21E-09 |
| ACTL6B | 0.565 | 1.40E-09 |
| GFAP | 0.565 | 1.33E-09 |
| OR8D1 | 0.565 | 1.38E-09 |
| ANKLE1 | 0.565 | 1.40E-09 |
| GP6 | 0.565 | 1.37E-09 |
| GSG1 | 0.565 | 1.35E-09 |
| EFCAB5 | 0.565 | 1.37E-09 |
| PYDC1 | 0.565 | 1.31E-09 |
| MYL10 | 0.565 | 1.31E-09 |
| CTD-2501B8.1 | 0.565 | 1.37E-09 |
| SLC22A14 | 0.565 | 1.40E-09 |
| C1orf141 | 0.565 | 1.38E-09 |
| TMPRSS3 | 0.564 | 1.48E-09 |
| HAP1 | 0.564 | 1.48E-09 |
| SPP2 | 0.564 | 1.51E-09 |
| ACER1 | 0.564 | 1.44E-09 |
| OR2L3 | 0.564 | 1.52E-09 |
| POLN | 0.564 | 1.46E-09 |
| ZNF891 | 0.563 | 1.56E-09 |
| ATAD3C | 0.563 | 1.59E-09 |
| L1TD1 | 0.563 | 1.61E-09 |
| CLEC4M | 0.563 | 1.66E-09 |
| FBXW10 | 0.563 | 1.60E-09 |
| ARHGAP8 | 0.563 | 1.63E-09 |
| CCDC60 | 0.562 | 1.79E-09 |
| OR1J1 | 0.562 | 1.76E-09 |
| C1orf158 | 0.561 | 1.87E-09 |
| TMC5 | 0.561 | 1.90E-09 |
| CYP2C9 | 0.561 | 1.91E-09 |
| STPG2 | 0.561 | 1.89E-09 |
| 14-Sep | 0.561 | 1.88E-09 |
| UGT2A1 | 0.56 | 1.96E-09 |
| GJB7 | 0.56 | 2.08E-09 |
| SPINK8 | 0.56 | 2.10E-09 |
| TMEM105 | 0.56 | 2.08E-09 |
| RNF223 | 0.56 | 1.96E-09 |
| ASGR1 | 0.56 | 1.99E-09 |
| MYH7B | 0.559 | 2.19E-09 |
| CTD-3105H18.18 | 0.558 | 2.41E-09 |
| RGS7 | 0.558 | 2.38E-09 |
| TRIML2 | 0.558 | 2.45E-09 |
| B3GNT6 | 0.558 | 2.40E-09 |
| POF1B | 0.558 | 2.36E-09 |
| UGT2B11 | 0.556 | 2.77E-09 |
| NANOS2 | 0.556 | 2.86E-09 |
| BSPRY | 0.556 | 2.79E-09 |
| OPRD1 | 0.556 | 2.70E-09 |
| MFSD4 | 0.556 | 2.81E-09 |
| SLC25A34 | 0.555 | 3.14E-09 |
| KRT26 | 0.555 | 2.96E-09 |
| CYLC1 | 0.555 | 3.13E-09 |
| LRP2BP | 0.555 | 3.06E-09 |
| GPR12 | 0.554 | 3.38E-09 |
| OOSP2 | 0.554 | 3.26E-09 |
| IZUMO2 | 0.554 | 3.33E-09 |
| VWC2 | 0.554 | 3.38E-09 |
| GJD2 | 0.554 | 3.23E-09 |
| ADAM2 | 0.554 | 3.34E-09 |
| ATXN3 | 0.554 | 3.24E-09 |
| VWA3A | 0.553 | 3.50E-09 |
| TBC1D21 | 0.553 | 3.65E-09 |
| PCDHA10 | 0.553 | 3.52E-09 |
| SI | 0.553 | 3.43E-09 |
| CCDC122 | 0.553 | 3.66E-09 |
| C4BPB | 0.553 | 3.45E-09 |
| NAP1L6 | 0.552 | 3.75E-09 |
| PNPLA1 | 0.552 | 3.87E-09 |
| LCN12 | 0.552 | 3.73E-09 |
| 12-Sep | 0.552 | 3.81E-09 |
| TXK | 0.552 | 3.75E-09 |
| HMHB1 | 0.552 | 3.72E-09 |
| SAA2 | 0.552 | 3.95E-09 |
| NR1I3 | 0.552 | 3.79E-09 |
| HTRA4 | 0.552 | 3.72E-09 |
| KRT15 | 0.552 | 3.91E-09 |
| MIPOL1 | 0.551 | 4.07E-09 |
| SAXO1 | 0.551 | 4.22E-09 |
| ZNF556 | 0.551 | 4.04E-09 |
| TRPV5 | 0.551 | 4.23E-09 |
| AP3B2 | 0.55 | 4.59E-09 |
| APOF | 0.55 | 4.36E-09 |
| BEST2 | 0.55 | 4.55E-09 |
| ZSCAN4 | 0.55 | 4.30E-09 |
| CDH12 | 0.55 | 4.55E-09 |
| LIX1 | 0.55 | 4.56E-09 |
| OR10H5 | 0.549 | 4.91E-09 |
| FUT2 | 0.549 | 4.94E-09 |
| DEFB118 | 0.549 | 4.87E-09 |
| GOLGA6L6 | 0.549 | 4.91E-09 |
| OR6C6 | 0.549 | 4.75E-09 |
| CLNK | 0.548 | 5.16E-09 |
| CHRNA2 | 0.548 | 5.16E-09 |
| OR2L2 | 0.548 | 5.01E-09 |
| FABP7 | 0.548 | 5.40E-09 |
| TYR | 0.547 | 5.77E-09 |
| TAAR8 | 0.547 | 5.80E-09 |
| C4orf17 | 0.547 | 5.79E-09 |
| PIWIL3 | 0.547 | 5.54E-09 |
| GADL1 | 0.547 | 5.60E-09 |
| OTOG | 0.547 | 5.52E-09 |
| FOXL2NB | 0.547 | 5.69E-09 |
| SLC14A2 | 0.546 | 6.00E-09 |
| POTEM | 0.546 | 6.23E-09 |
| IRGM | 0.546 | 6.16E-09 |
| SPTLC3 | 0.546 | 5.98E-09 |
| DSG3 | 0.546 | 5.85E-09 |
| SERPINA10 | 0.545 | 6.81E-09 |
| CDH18 | 0.545 | 6.76E-09 |
| C15orf43 | 0.545 | 6.62E-09 |
| KCNE1 | 0.545 | 6.40E-09 |
| NCCRP1 | 0.545 | 6.31E-09 |
| KCNH5 | 0.545 | 6.57E-09 |
| CATSPER2 | 0.545 | 6.56E-09 |
| C3orf49 | 0.544 | 7.01E-09 |
| OR52A1 | 0.544 | 7.04E-09 |
| HPSE2 | 0.544 | 7.09E-09 |
| CADPS | 0.544 | 7.25E-09 |
| DNAJB13 | 0.543 | 7.82E-09 |
| SLC24A2 | 0.543 | 7.92E-09 |
| ESR2 | 0.543 | 7.50E-09 |
| KLHL33 | 0.543 | 7.35E-09 |
| SLC3A1 | 0.543 | 7.48E-09 |
| LGALS4 | 0.543 | 7.40E-09 |
| OR52E4 | 0.543 | 7.63E-09 |
| GALR1 | 0.543 | 7.72E-09 |
| TVP23A | 0.543 | 7.36E-09 |
| TMPRSS7 | 0.542 | 8.04E-09 |
| CHAT | 0.542 | 8.52E-09 |
| RUNDC3A | 0.541 | 8.88E-09 |
| FNDC7 | 0.541 | 9.10E-09 |
| TMEM82 | 0.541 | 8.73E-09 |
| IFNA16 | 0.541 | 8.59E-09 |
| ZNF670-ZNF695 | 0.541 | 9.18E-09 |
| TSPYL6 | 0.54 | 9.31E-09 |
| SYCP1 | 0.54 | 9.54E-09 |
| FAM81B | 0.54 | 9.25E-09 |
| UGT1A10 | 0.54 | 9.90E-09 |
| OR4F17 | 0.54 | 9.66E-09 |
| ALS2CR12 | 0.54 | 9.27E-09 |
| P2RX5-TAX1BP3 | 0.54 | 9.63E-09 |
| FRMPD1 | 0.54 | 9.21E-09 |
| FAM83F | 0.539 | 1.03E-08 |
| DCX | 0.539 | 9.93E-09 |
| CYP8B1 | 0.539 | 9.95E-09 |
| APOB | 0.539 | 1.05E-08 |
| SCN2A | 0.539 | 1.05E-08 |
| SPAM1 | 0.539 | 1.03E-08 |
| ADGB | 0.539 | 1.07E-08 |
| AHSG | 0.538 | 1.10E-08 |
| C1orf234 | 0.538 | 1.13E-08 |
| DPRX | 0.538 | 1.08E-08 |
| CYP3A5 | 0.538 | 1.10E-08 |
| GABRG3 | 0.538 | 1.08E-08 |
| RSPH10B | 0.538 | 1.09E-08 |
| LRRC36 | 0.538 | 1.11E-08 |
| LYZL2 | 0.538 | 1.14E-08 |
| ADAD1 | 0.538 | 1.09E-08 |
| ZPBP | 0.538 | 1.12E-08 |
| POTEC | 0.537 | 1.18E-08 |
| GCKR | 0.537 | 1.17E-08 |
| PHOX2B | 0.537 | 1.23E-08 |
| LRRC10 | 0.537 | 1.20E-08 |
| ALPP | 0.537 | 1.24E-08 |
| PLG | 0.537 | 1.24E-08 |
| LMNTD1 | 0.536 | 1.29E-08 |
| KLRK1 | 0.536 | 1.30E-08 |
| B3GAT1 | 0.536 | 1.28E-08 |
| ACKR2 | 0.536 | 1.27E-08 |
| OR4F15 | 0.536 | 1.26E-08 |
| LAMB4 | 0.536 | 1.33E-08 |
| ANKRD20A4 | 0.536 | 1.29E-08 |
| KCNT1 | 0.536 | 1.31E-08 |
| GABRR3 | 0.536 | 1.28E-08 |
| CCDC129 | 0.536 | 1.29E-08 |
| FAM228B | 0.535 | 1.36E-08 |
| C16orf78 | 0.535 | 1.41E-08 |
| ZNF66 | 0.535 | 1.35E-08 |
| MCHR2 | 0.535 | 1.36E-08 |
| SCNN1G | 0.535 | 1.38E-08 |
| SLC34A2 | 0.535 | 1.43E-08 |
| DCST1 | 0.535 | 1.35E-08 |
| OR10D3 | 0.535 | 1.35E-08 |
| CYP17A1 | 0.534 | 1.52E-08 |
| PSG6 | 0.534 | 1.48E-08 |
| DQX1 | 0.534 | 1.51E-08 |
| OR52N5 | 0.534 | 1.44E-08 |
| U51561.1 | 0.533 | 1.60E-08 |
| SGSM1 | 0.533 | 1.60E-08 |
| GPR26 | 0.533 | 1.57E-08 |
| ABO | 0.533 | 1.56E-08 |
| C11orf40 | 0.532 | 1.73E-08 |
| SHCBP1L | 0.532 | 1.71E-08 |
| FAM227A | 0.532 | 1.71E-08 |
| DNAAF1 | 0.532 | 1.78E-08 |
| CACNA1A | 0.531 | 1.91E-08 |
| RP11-506B6.7 | 0.531 | 1.84E-08 |
| ASZ1 | 0.531 | 1.91E-08 |
| CAPN12 | 0.53 | 2.06E-08 |
| FCRL4 | 0.53 | 1.99E-08 |
| GFRAL | 0.53 | 1.98E-08 |
| ZBP1 | 0.53 | 2.07E-08 |
| C1orf146 | 0.529 | 2.17E-08 |
| UTS2B | 0.529 | 2.16E-08 |
| GPR83 | 0.529 | 2.11E-08 |
| CD160 | 0.529 | 2.18E-08 |
| CLEC4E | 0.529 | 2.10E-08 |
| HRG | 0.528 | 2.32E-08 |
| HTN3 | 0.528 | 2.39E-08 |
| STARD9 | 0.528 | 2.26E-08 |
| GSG1L2 | 0.528 | 2.35E-08 |
| SLC24A4 | 0.528 | 2.37E-08 |
| SLC6A4 | 0.527 | 2.46E-08 |
| PPP2R2B | 0.527 | 2.55E-08 |
| OR10A3 | 0.527 | 2.40E-08 |
| PDE3B | 0.527 | 2.42E-08 |
| TDRD12 | 0.527 | 2.50E-08 |
| GABRA2 | 0.527 | 2.57E-08 |
| OR6B1 | 0.527 | 2.41E-08 |
| CCDC158 | 0.526 | 2.67E-08 |
| MYT1L | 0.526 | 2.71E-08 |
| HAL | 0.526 | 2.64E-08 |
| STMN4 | 0.526 | 2.70E-08 |
| C4orf26 | 0.526 | 2.70E-08 |
| STX1B | 0.526 | 2.74E-08 |
| SFTA3 | 0.526 | 2.63E-08 |
| WDR87 | 0.526 | 2.70E-08 |
| CTB-50L17.14 | 0.526 | 2.74E-08 |
| MYBPC3 | 0.526 | 2.73E-08 |
| KDF1 | 0.526 | 2.59E-08 |
| C2orf80 | 0.525 | 2.82E-08 |
| MC5R | 0.525 | 2.96E-08 |
| UNC80 | 0.525 | 2.97E-08 |
| KRT33B | 0.525 | 2.95E-08 |
| TLR10 | 0.525 | 2.85E-08 |
| SERPINB13 | 0.525 | 2.78E-08 |
| TECRL | 0.524 | 3.12E-08 |
| ZG16 | 0.524 | 3.05E-08 |
| LRRC71 | 0.524 | 3.14E-08 |
| SHH | 0.524 | 3.08E-08 |
| TTC21A | 0.524 | 3.06E-08 |
| ZFR2 | 0.523 | 3.42E-08 |
| CACNG8 | 0.523 | 3.26E-08 |
| FAM184B | 0.523 | 3.26E-08 |
| UGT2B15 | 0.523 | 3.36E-08 |
| TMEM266 | 0.523 | 3.27E-08 |
| PKD2L2 | 0.522 | 3.57E-08 |
| MUC21 | 0.522 | 3.60E-08 |
| FAM231A | 0.522 | 3.42E-08 |
| KYNU | 0.522 | 3.59E-08 |
| PRSS55 | 0.522 | 3.66E-08 |
| ACSBG1 | 0.522 | 3.65E-08 |
| AMER3 | 0.522 | 3.66E-08 |
| ERICH6B | 0.522 | 3.52E-08 |
| L3MBTL1 | 0.522 | 3.49E-08 |
| NCR1 | 0.522 | 3.46E-08 |
| TMEM154 | 0.521 | 3.93E-08 |
| CR2 | 0.521 | 3.79E-08 |
| WBP2NL | 0.521 | 3.70E-08 |
| GJD4 | 0.521 | 3.82E-08 |
| GDPD2 | 0.521 | 3.75E-08 |
| AQP7 | 0.521 | 3.83E-08 |
| FUT9 | 0.521 | 3.79E-08 |
| BCO1 | 0.521 | 3.82E-08 |
| KBTBD12 | 0.521 | 3.73E-08 |
| HIF3A | 0.521 | 3.85E-08 |
| CFAP74 | 0.521 | 3.88E-08 |
| MGAT4D | 0.52 | 4.10E-08 |
| GDF5OS | 0.52 | 4.15E-08 |
| CATSPER4 | 0.52 | 4.06E-08 |
| CCDC33 | 0.52 | 3.95E-08 |
| ERVFRD-1 | 0.52 | 3.94E-08 |
| OR5C1 | 0.519 | 4.29E-08 |
| TTLL6 | 0.519 | 4.53E-08 |
| CDH16 | 0.519 | 4.26E-08 |
| SERPINB2 | 0.519 | 4.51E-08 |
| RBFOX1 | 0.519 | 4.34E-08 |
| CABP5 | 0.519 | 4.30E-08 |
| SV2B | 0.519 | 4.35E-08 |
| LRRC19 | 0.519 | 4.38E-08 |
| UGT1A7 | 0.518 | 4.76E-08 |
| TMEM95 | 0.518 | 4.55E-08 |
| PADI6 | 0.518 | 4.57E-08 |
| SH2D6 | 0.518 | 4.63E-08 |
| CSN3 | 0.518 | 4.77E-08 |
| CBWD7 | 0.517 | 5.20E-08 |
| RFX4 | 0.517 | 5.17E-08 |
| FSD2 | 0.517 | 4.96E-08 |
| TESPA1 | 0.517 | 5.09E-08 |
| CLDN19 | 0.516 | 5.28E-08 |
| CYP46A1 | 0.516 | 5.37E-08 |
| KRTDAP | 0.516 | 5.42E-08 |
| TEX35 | 0.516 | 5.52E-08 |
| MGAM | 0.516 | 5.24E-08 |
| CACNA1F | 0.516 | 5.23E-08 |
| EFCAB9 | 0.516 | 5.32E-08 |
| DDX4 | 0.515 | 5.76E-08 |
| ZNF169 | 0.515 | 5.62E-08 |
| SLC4A5 | 0.515 | 5.93E-08 |
| POU2AF1 | 0.514 | 6.18E-08 |
| KSR2 | 0.514 | 6.19E-08 |
| FER1L5 | 0.514 | 6.06E-08 |
| C12orf54 | 0.514 | 6.00E-08 |
| FAM106A | 0.514 | 6.32E-08 |
| MTRNR2L3 | 0.514 | 6.23E-08 |
| COL20A1 | 0.514 | 6.09E-08 |
| RBPJL | 0.514 | 6.13E-08 |
| MORN3 | 0.514 | 6.41E-08 |
| ENPP3 | 0.514 | 6.27E-08 |
| GPR63 | 0.514 | 6.05E-08 |
| NFASC | 0.513 | 6.42E-08 |
| ELAVL4 | 0.513 | 6.65E-08 |
| TMEM211 | 0.513 | 6.81E-08 |
| OR8S1 | 0.513 | 6.73E-08 |
| ADCY10 | 0.513 | 6.70E-08 |
| LGALS14 | 0.513 | 6.78E-08 |
| DUOX2 | 0.513 | 6.49E-08 |
| C2orf54 | 0.513 | 6.56E-08 |
| DLGAP1 | 0.512 | 6.86E-08 |
| DGKB | 0.512 | 7.26E-08 |
| LRRIQ1 | 0.512 | 7.02E-08 |
| MYCBPAP | 0.512 | 7.02E-08 |
| TMPRSS11A | 0.512 | 7.31E-08 |
| RP1L1 | 0.512 | 7.06E-08 |
| FBXO24 | 0.511 | 7.41E-08 |
| SH3TC2 | 0.511 | 7.64E-08 |
| CYP4A22 | 0.511 | 7.71E-08 |
| CA1 | 0.511 | 7.45E-08 |
| CYP4F2 | 0.511 | 7.50E-08 |
| SLC35F4 | 0.511 | 7.55E-08 |
| PSG3 | 0.51 | 8.04E-08 |
| ALDH1L1 | 0.51 | 8.08E-08 |
| NPFFR1 | 0.509 | 8.43E-08 |
| C1orf186 | 0.509 | 8.54E-08 |
| IL3 | 0.509 | 8.96E-08 |
| AARD | 0.509 | 8.55E-08 |
| IAPP | 0.509 | 8.66E-08 |
| PLPPR1 | 0.509 | 8.63E-08 |
| OR2J3 | 0.508 | 9.12E-08 |
| EBLN2 | 0.508 | 9.06E-08 |
| GCNT7 | 0.508 | 9.18E-08 |
| ADGRE3 | 0.508 | 9.21E-08 |
| LRG1 | 0.507 | 9.97E-08 |
| TRPM6 | 0.507 | 9.99E-08 |
| AANAT | 0.507 | 9.84E-08 |
| CNPY1 | 0.506 | 1.04E-07 |
| NOX3 | 0.506 | 1.08E-07 |
| C6orf10 | 0.506 | 1.06E-07 |
| MAGEB10 | 0.506 | 1.05E-07 |
| CTCFL | 0.506 | 1.06E-07 |
| CCL22 | 0.506 | 1.04E-07 |
| OR4F6 | 0.505 | 1.11E-07 |
| HHLA2 | 0.505 | 1.14E-07 |
| PRODH | 0.505 | 1.16E-07 |
| RP11-235E17.2 | 0.505 | 1.13E-07 |
| OR6N2 | 0.505 | 1.10E-07 |
| CASC1 | 0.504 | 1.20E-07 |
| C4orf22 | 0.504 | 1.20E-07 |
| FGF9 | 0.504 | 1.24E-07 |
| SRRM4 | 0.504 | 1.18E-07 |
| ZC3H12D | 0.504 | 1.20E-07 |
| PMP2 | 0.504 | 1.20E-07 |
| TMEM45B | 0.504 | 1.20E-07 |
| FGB | 0.503 | 1.28E-07 |
| APOH | 0.503 | 1.31E-07 |
| ATP13A5 | 0.503 | 1.27E-07 |
| SLC6A14 | 0.503 | 1.29E-07 |
| ZFP42 | 0.503 | 1.34E-07 |
| ZAN | 0.503 | 1.26E-07 |
| OR51V1 | 0.503 | 1.35E-07 |
| WNT8A | 0.503 | 1.32E-07 |
| CELA3A | 0.502 | 1.42E-07 |
| AWAT1 | 0.502 | 1.42E-07 |
| DEPDC4 | 0.502 | 1.37E-07 |
| CRHR2 | 0.502 | 1.37E-07 |
| ASIC4 | 0.501 | 1.51E-07 |
| BTNL8 | 0.501 | 1.47E-07 |
| OR5AP2 | 0.501 | 1.50E-07 |
| PRLHR | 0.501 | 1.45E-07 |
| SPINK9 | 0.501 | 1.47E-07 |
| IL17C | 0.5 | 1.60E-07 |
| NPIPB4 | 0.5 | 1.60E-07 |
| CORO2A | 0.5 | 1.57E-07 |
| CEACAM6 | 0.5 | 1.60E-07 |
| FRMPD2 | 0.5 | 1.57E-07 |
| UBAP1L | 0.5 | 1.56E-07 |
| KIAA1328 | 0.5 | 1.61E-07 |
| TBC1D32 | 0.5 | 1.54E-07 |
